# Maternal Setdb1 Is Required for Meiotic Progression and Preimplantation Development in Mouse

**DOI:** 10.1371/journal.pgen.1005970

**Published:** 2016-04-12

**Authors:** Jeesun Kim, Hongbo Zhao, Jiameng Dan, Soojin Kim, Swanand Hardikar, Debra Hollowell, Kevin Lin, Yue Lu, Yoko Takata, Jianjun Shen, Taiping Chen

**Affiliations:** 1 Department of Epigenetics and Molecular Carcinogenesis, The University of Texas MD Anderson Cancer Center, Smithville, Texas, United States of America; 2 Center for Cancer Epigenetics, The University of Texas MD Anderson Cancer Center, Smithville, Texas, United States of America; 3 Shanghai Key Laboratory of Female Reproductive Endocrine Related Diseases, Hospital and Institute of Obstetrics and Gynecology, Fudan University, Shanghai, People’s Republic of China; 4 The University of Texas Graduate School of Biomedical Sciences at Houston, Houston, Texas, United States of America; CNRS/UPMC, FRANCE

## Abstract

Oocyte meiotic progression and maternal-to-zygote transition are accompanied by dynamic epigenetic changes. The functional significance of these changes and the key epigenetic regulators involved are largely unknown. Here we show that Setdb1, a lysine methyltransferase, controls the global level of histone H3 lysine 9 di-methyl (H3K9me2) mark in growing oocytes. Conditional deletion of *Setdb1* in developing oocytes leads to meiotic arrest at the germinal vesicle and meiosis I stages, resulting in substantially fewer mature eggs. Embryos derived from these eggs exhibit severe defects in cell cycle progression, progressive delays in preimplantation development, and degeneration before reaching the blastocyst stage. Rescue experiments by expressing wild-type or inactive Setdb1 in *Setdb1*-deficient oocytes suggest that the catalytic activity of Setdb1 is essential for meiotic progression and early embryogenesis. Mechanistically, up-regulation of Cdc14b, a dual-specificity phosphatase that inhibits meiotic progression, greatly contributes to the meiotic arrest phenotype. *Setdb1* deficiency also leads to derepression of transposons and increased DNA damage in oocytes, which likely also contribute to meiotic defects. Thus, *Setdb1* is a maternal-effect gene that controls meiotic progression and is essential for early embryogenesis. Our results uncover an important link between the epigenetic machinery and the major signaling pathway governing meiotic progression.

## Author Summary

During oogenesis, oocytes accumulate transcripts and proteins that support meiotic maturation and early embryogenesis. Although a number of such maternal-effect factors have been identified, our knowledge about the molecular machinery that drives meiotic progression and maternal-to-zygotic transition is still limited. In particular, the functional significance of epigenetic changes, which accompany meiotic maturation and early embryogenesis, and the key epigenetic regulators involved are largely unknown. Here, we identify Setdb1, a lysine methyltransferase specific for the repressive histone H3 lysine 9 (H3K9) methylation, as a maternal-effect factor that is essential for meiotic progression in oocytes and mitotic cell divisions in early embryos in mouse. We show that Setdb1 is highly expressed in growing oocytes and directly represses the expression Cdc14b, a phosphatase that inhibits meiotic progression. Setdb1 is also required to repress retrotransposons and maintain genomic stability in oocytes. Embryos derived from Setdb1-depleted oocytes show severe defects in cell cycle progression, progressive delays in preimplantation development, and degeneration before reaching the blastocyst stage. The roles of Setdb1 in meiotic progression and preimplantation development require its catalytic activity. Our findings demonstrate that Setdb1 is an important regulator of Cdc14b, thus uncovering a molecular link between the epigenetic machinery and the major signaling pathway that drives meiotic progression.

## Introduction

Mammalian development begins with fertilization, when the haploid sperm and egg fuse to form the diploid zygote. Although both gametes have equal genetic contributions to the offspring, the early embryo is almost entirely dependent on the egg for the supply of subcellular organelles and macromolecules for initial survival and development [1]. These maternal components are encoded by maternal-effect genes, which are transcribed in oocytes and their products (RNA or protein) are present in early embryos before expression of zygotic genes is initiated. Since the identification of the first mammalian maternal-effect genes in 2000 [2,3], multiple such genes have been reported [4]. Genetic studies in mice suggest important roles of maternal-effect genes in developmental processes, including epigenetic reprogramming, zygotic genome activation (ZGA), and cell specification [4]. Despite the progress, the molecular machinery and regulatory mechanisms involved in meiotic progression and maternal-to-zygotic transition are not well understood.

In females, meiosis is initiated during fetal development, and oocytes are arrested at prophase I around the time of birth. During subsequent folliculogenesis, the diameters of oocytes increase dramatically, even though prophase I arrest remains in effect. Transcription of the maternal genome occurs predominantly during oocyte growth. Some transcripts are translated immediately into proteins, and others are stored for later activation [1]. Prophase I arrest is sustained until puberty when luteinizing hormone (LH) induces resumption of meiosis. The first visible sign of meiotic resumption is nuclear envelope (called germinal vesicle, GV) breakdown (GVBD). Following GVBD, a metaphase I spindle forms and stable microtubule-kinetochore interactions are established in all chromosome bivalents before proceeding to anaphase I and telophase I. After completion of meiosis I (MI), as indicated by the extrusion of the first polar body, oocytes enter directly into meiosis II without an intervening S-phase and arrest again at metaphase II (Met II). Fertilization triggers resumption and completion of meiosis II [5].

Meiotic progression is governed by the maturation-promoting factor (MPF), which consists of cyclin-dependent kinase 1 (Cdk1, also known as Cdc2) and a regulatory subunit Cyclin B1. In prophase I-arrested GV oocytes, Cdk1 is inactivated by Wee2-mediated phosphorylation on Thr14 and Tyr15, and Cyclin B1 is constantly degraded by the anaphase-promoting complex/cyclosome (APC/C), a multisubunit E3 ubiquitin ligase. The preovulatory LH surge triggers meiotic resumption by alleviating Cdk1 phosphorylation and inducing Cyclin B1 accumulation [6].

Other kinases and phosphatases also participate in meiotic progression. These include Cdc14b, a highly conserved dual-specificity phosphatase that counteracts the activity of Cdk1 [7]. In somatic cells, Cdc14b has been implicated in multiple cellular processes, including nuclear organization, spindle assembly, mitotic exit, and DNA damage response and repair [7]. In oocytes, Cdc14b is a negative regulator of meiotic progression. Oocytes overexpressing Cdc14b are significantly delayed in resuming meiosis and fail to progress to the Met II stage. Conversely, depletion of Cdc14b in GV oocytes leads to premature meiotic resumption [8]. Cdc14b is also present in preimplantation embryos. Overexpression of Cdc14b in 1-cell embryos has been shown to cause mitotic arrest and inhibit ZGA [9]. These findings suggest that proper regulation of Cdc14b expression is important for meiosis and early embryogenesis. However, little is known about how Cdc14b expression is regulated.

Meiotic progression and early embryogenesis are accompanied by drastic chromatin remodeling and epigenetic reprogramming [10,11]. Epigenetic events, including posttranslational modifications of histones, are believed to play crucial roles during meiosis and embryogenesis. Indeed, progress has been made in documenting epigenetic states in these processes. For example, during meiotic maturation, histone H3 and H4 are globally deacetylated, whereas H3 lysine 9 di- and tri-methyl (H3K9me2/me3) marks remain constantly high [12,13]. However, the functional relevance of epigenetic events and the key epigenetic regulators involved during oogenesis and early embryogenesis remain largely unknown.

Setdb1, also known as Eset and KMT1E, is a lysine methyltransferase (KMT) specific for the repressive histone H3 lysine 9 di- and tri-methyl (H3K9me2/me3) marks [14,15]. It is associated with transcriptional repression of euchromatic genes and maintenance of heterochromatin structure [14,15,16]. Recent evidence suggests that Setdb1 also plays a critical role in silencing retrotransposons in undifferentiated embryonic stem (ES) cells, as well as in early embryos and primordial germ cells (PGCs), where DNA methylation levels are low due to epigenetic reprogramming [17,18]. DNA methylation is required for retrotransposon silencing in somatic cells [19]. *Setdb1* is an evolutionally conserved gene. Its *Drosophila* ortholog *dSetdb1* (also known as *dEset* and *Eggless*) is involved in multiple developmental processes, including oogenesis [20,21,22]. Mouse embryos lacking Setdb1 die at the peri-implantation stage (around 3.5–5.5 days post coitum (dpc)) [23], which is significantly earlier than the phenotypes of mice deficient for other H3K9 KMTs, such as Suv39h1/Suv39h2 (developmental defects after ~12.5 dpc) [24] and G9a (lethality at ~9.5 dpc) [25]. Setdb1 is present at high levels in oocytes and zygotes and persists through preimplantation development [26,27]. However, expression of zygotic *Setdb1* is undetectable until the blastocyst stage [23,26]. These observations suggest that maternal Setdb1 may play important roles in oogenesis and/or early embryogenesis.

Here, we show that maternal Setdb1 is essential for meiotic progression in oocytes and mitotic cell cycle progression in early embryos. Conditional deletion of *Setdb1* in growing oocytes leads to severe defects in meiotic resumption and maturation, largely due to up-regulation of Cdc14b, resulting in the production of considerably fewer Met II oocytes. Although these Met II oocytes are fertilizable, the resulting embryos display impaired cell cycle progression, progressive delays in preimplantation development, and degeneration before reaching the blastocyst stage. The functions of Setdb1 in these processes require its catalytic activity. Our work identifies *Setdb1* as a maternal-effect gene essential for fertility and uncovers a functional link between Setdb1 and the signaling pathway governing meiotic progression.

## Results

### Setdb1 is expressed and controls global H3K9me2 levels in growing oocytes

**Fig 1:**
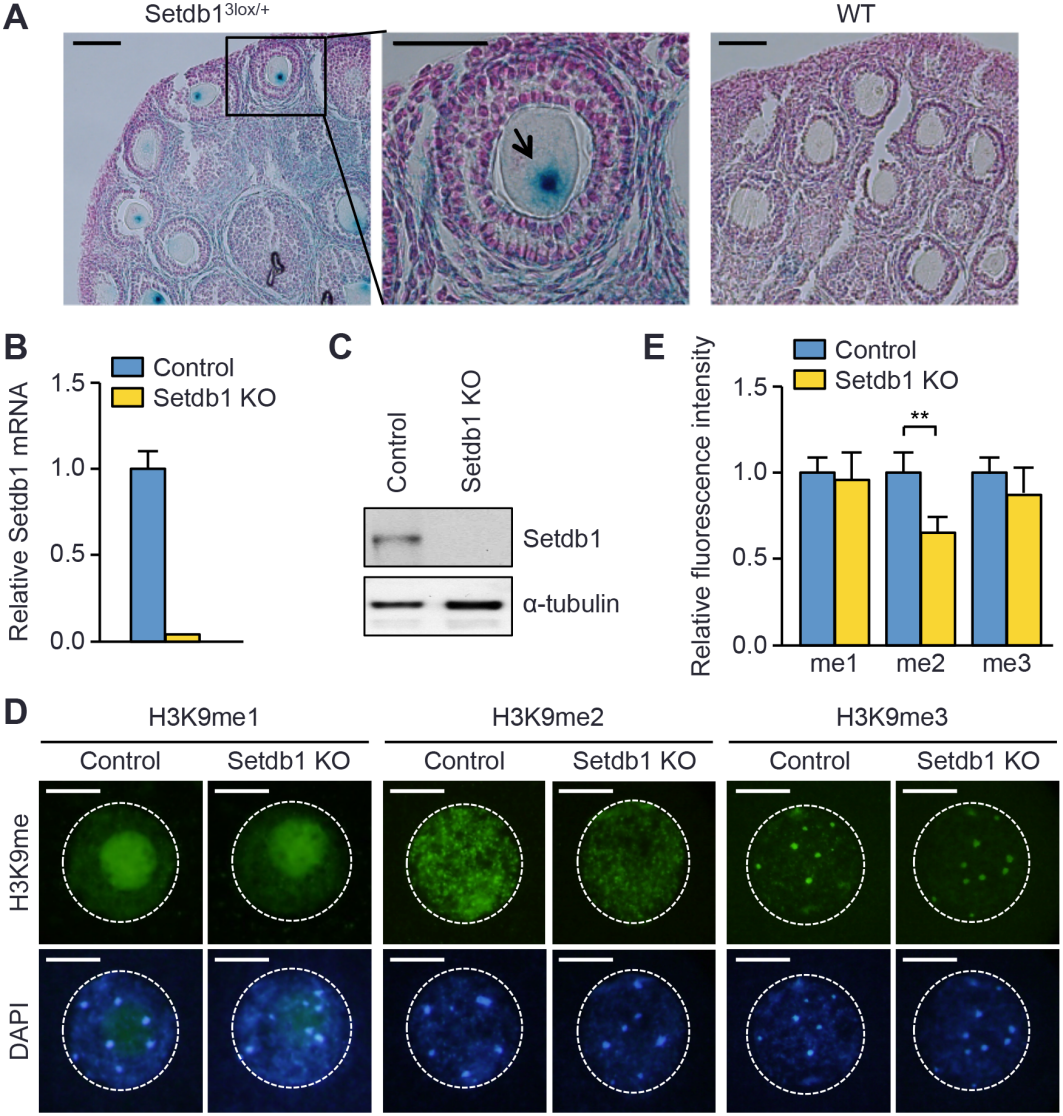
Setdb1 is expressed and controls global H3K9me2 in growing oocytes. **(A)** X-gal staining of paraffin-embedded sections of ovaries from 4-week-old *Setdb1*^*3lox/+*^ or wild-type (WT) mice. One follicle is enlarged, and the oocyte nucleus is indicated by an arrow. Scale bars, 50 μm. **(B)** qRT-PCR analysis of *Setdb1* transcript. Shown are relative levels of *Setdb1* mRNA in control and *Setdb1* KO GV oocytes (mean ± SEM of triplicate assays). **(C)** Western blot analysis of Setdb1 in control and *Setdb1* KO GV oocytes, with α-tubulin as a loading control. Each lane contains 150 GV oocytes. **(D, E)** IF analysis of H3K9 methylation in oocytes. Fully-grown GV oocytes harvested from control and *Setdb1* KO mice were immunostained with antibodies specific for H3K9 mono-, di- and tri-methyl (H3K9me1, H3K9me2, and H3K9me3) marks and counterstained with DAPI. **(D)** Representative images of H3K9me1, H3K9me2, and H3K9me3 marks (green) and DAPI (blue). The nuclei of the oocytes are circled. Note that H3K9me1 is enriched in the nucleoli, H3K9me2 exhibits a punctate staining pattern throughout the nuclei, and H3K9me3 is enriched in constitutive heterochromatin. Scale bars, 10 μm. **(E)** Quantification of fluorescence intensity of H3K9me1, H3K9me2, and H3K9me3 marks in oocytes. Fifteen oocytes for each genotype were stained by each antibody, and the data are presented as the mean ± SEM. Statistical comparisons were made using unpaired t-test. ** P < 0.01.

Although previous work detected *Setdb1* transcript and protein in isolated oocytes [23,26,27], its expression during oogenesis has not been characterized. We examined *Setdb1* expression in the ovary, taking advantage of the availability of the *Setdb1*^*3lox*^ allele (schematically shown in S1 Fig), which expresses the *lacZ* -galactosidase reporter under the control of the regulatory elements of endogenous *Setdb1* [28]. X-gal (5-bromo-4-chloro-3-indoyl-D-galactoside) staining of paraffin-embedded sections of ovaries from 4-week-old *Setdb1*^*3lox/+*^ (heterozygous) mice detected strong *lacZ* signal in growing oocytes, with little staining in granulosa cells (Fig 1A). The *lacZ* signal was specific, because no staining was observed in ovaries from wild-type (WT) mice (Fig 1A). Quantitative RT-PCR (qRT-PCR) and Western blot analyses confirmed the presence of *Setdb1* transcript and protein in fully-grown GV oocytes (Fig 1B and 1C). These results demonstrated that *Setdb1* is actively transcribed and translated during oocyte growth.

Zygotic Setdb1 is essential for embryonic development [23]. To determine the role of maternal Setdb1, we conditionally deleted exon 16 of *Setdb1* in oocytes. Deletion of exon 16 would remove 209 amino acids in the catalytic bifurcated SET domain and create a stop codon, thus resulting in a functionally null allele [28]. To maximize the deletion efficiency, heterozygous mice bearing a null allele, *Setdb1*^*1lox*^, were first crossed with Zp3-Cre transgenic mice, which express the Cre recombinase exclusively in growing oocytes [29], and the resulting *Setdb1*^*1lox/+*^*/Zp3-Cre*^*+*^ male mice were then crossed with female mice homozygous for the *Setdb1* conditional allele, *Setdb1*^*2lox*^ (see S1 Fig for breeding scheme). *Setdb1*^*2lox/1lox*^*/Zp3-Cre*^*+*^ female mice were used as the experimental group and, for simplicity, will be referred to as *Setdb1* knockout (KO) mice hereafter. Mice of the other genotypes (*Setdb1*^*2lox/+*^*/Zp3-Cre*^*-*^, *Setdb1*^*2lox/1lox*^*/Zp3-Cre*^*-*^, and *Setdb1*^*2lox/+*^*/Zp3-Cre*^*+*^) produced from the breeding scheme showed no defect in fertility and other phenotypic abnormalities, and *Setdb1*^*2lox/+*^*/Zp3-Cre*^*-*^ female mice were used as the control group. Genotypes were determined by PCR analysis of tail DNA samples (see S1 Fig for examples). qRT-PCR and Western blot analyses confirmed the complete elimination of *Setdb1* transcript and protein in *Setdb1* KO GV oocytes (Fig 1B and 1C).

Consistent with previous findings that Setdb1 is the predominant H3K9 KMT in oocytes and it catalyzes di- and tri-methylation [14,27], immunofluorescence (IF) and immunohistochemistry (IHC) analyses revealed that the global level of H3K9me2 significantly decreased and that of H3K9me3 slightly decreased in *Setdb1* KO oocytes, whereas the levels of H3K9me1 and H3K4me2 showed no alterations (Fig 1D and 1E and S2 Fig). These results indicated that, in oocytes, Setdb1 controls the global level of H3K9me2 mark and its effect on H3K9me3 could be loci-specific.

### *Setdb1* KO oocytes show severe defects in meiotic resumption and maturation

To determine the impact of maternal Setdb1 depletion on fertility, six *Setdb1* KO females were mated with WT males for 5 months. Although vaginal plugs were frequently observed, none of the mice produced pups, indicating that maternal Setdb1 is essential for fertility.

The infertility phenotype could be due to defects in oogenesis, embryogenesis, or both. We first examined *Setdb1* KO ovaries and found that they were morphologically and histologically indistinguishable from control ovaries, with the presence of follicles at various stages (S3 Fig). Fully-grown GV oocytes isolated from *Setdb1* KO mice also appeared normal in morphology and number (S3 Fig). These observations indicated that *Setdb1* depletion had no effect on folliculogenesis and oocyte growth.

**Fig 2:**
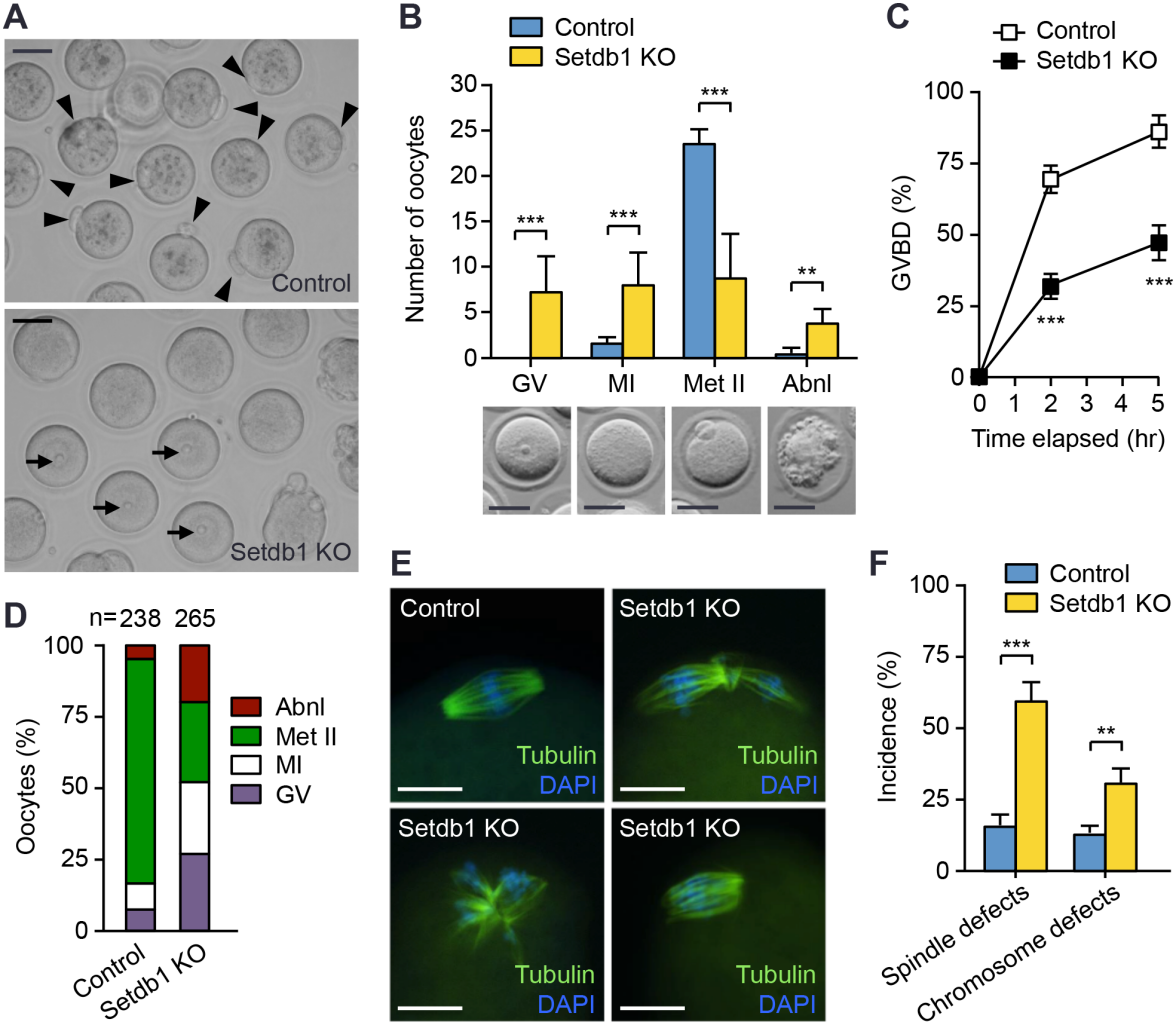
*Setdb1* KO oocytes show severe meiotic arrest. **(A, B)** Oocytes harvested from the oviducts of control and *Setdb1* KO mice at 16 hours post-hCG injection. **(A)** Representative bright-field microscope images of control and *Setdb1* KO oocytes. Arrowheads and arrows indicate the polar bodies (characteristic of Met II oocytes) and the prominent nucleoli (characteristic of GV oocytes), respectively. Scale bars, 50 μm. **(B)** Quantification of oocytes at different meiotic stages. Oocytes were classified as being GV arrested (GV), meiosis I (MI), metaphase II (Met II), or abnormal (Abnl, including those showing abnormal morphologies or undergoing degeneration). A representative oocyte of each type is shown at the bottom. Scale bars, 50 μm. Plotted are the average numbers of oocytes (mean ± SEM) at different stages from 12 control mice (306 oocytes in total) and 13 KO mice (364 oocytes in total). Statistical comparisons were made using unpaired t-test. **P < 0.01; ***P < 0.001. **(C, D)** *In vitro* oocyte maturation assays. Fully-grown GV oocytes from 6-week-old control and *Setdb1* KO mice were collected in M2 medium containing IBMX (200 μM) and, following IBMX washout, cultured in IBMX-free M16 medium. **(C)** The GVBD rates at 2 hours and 5 hours after IBMX removal. Plotted are data from 4 control mice (142 oocytes in total) and 4 KO mice (160 oocytes in total). Statistical comparisons were made using unpaired t-test. ***P < 0.001. **(D)** The percentages of oocytes at different meiotic stages after 20 hours of culture. Plotted are data from 6 control mice (238 oocytes in total) and 6 KO mice (265 oocytes in total). **(E, F)** Spindle and chromosome defects in *Setdb1* KO MI oocytes. Fully-grown GV oocytes harvested from control and *Setdb1* KO mice were cultured in maturation medium for 5 hours, and the oocytes were immunostained with α-tubulin antibody and DAPI to examine spindle and chromosome structures, respectively. **(E)** Representative IF images showing normal spindle (α-tubulin, green) and chromosome (DAPI, blue) morphologies in control MI oocytes and common abnormalities in KO MI oocytes. Spindle defects include dispersed, tread-like, non-bipolar, and multiple spindles. Chromosome defects include decondensed, lagging, and misaligned chromosomes. Scale bars, 25 μm. **(F)** Frequencies of spindle and chromosome defects (mean ± SEM) in MI oocytes. In total, 253 control MI oocytes and 232 KO MI oocytes were examined. Statistical comparisons were made using unpaired t-test. **P < 0.01; ***P < 0.001.

We then assessed whether *Setdb1* deficiency affected meiotic resumption and maturation. After superovulation, the vast majority (>90%) of oocytes collected from the oviducts of control mice, as expected, were arrested at the Met II stage, judged by the presence of a polar body. Although similar numbers of oocytes were recovered from the oviducts of superovulated *Setdb1* KO mice, much smaller fractions were at the Met II stage, varying from ~20% to ~60% in different litters. The rest was arrested at the GV stage (~15–40%), based on the presence of an intact GV, and at MI (~15–40%), as evidenced by the absence of both GV and polar body, or were abnormal (~10–20%) (Fig 2A and 2B). These observations suggested that a significant fraction of *Setdb1* KO oocytes failed to develop to the Met II stage before being released at ovulation.

To verify the meiotic arrest phenotype, we isolated GV oocytes and performed *in vitro* meiotic maturation assays. Fully-grown GV oocytes, when removed from their follicular environment, undergo spontaneous meiotic resumption, which can be reversibly inhibited by cyclic adenosine monophosphate (cAMP) phosphodiesterase inhibitors such as 3-isobutyl-1-methylxanthine (IBMX). Control and *Setdb1* KO GV oocytes were initially collected in IBMX-containing medium and then cultured in the absence of IBMX for various periods of time. Examination at 2 and 5 hours after IBMX removal revealed that *Setdb1* KO oocytes underwent GVBD significantly more slowly than control oocytes (Fig 2C). Following 20 hours of culture, ~90% of control oocytes resumed meiosis, and ~80% progressed to the Met II stage. In contrast, nearly 30% of *Setdb1* KO oocytes remained arrested at the GV stage, ~25% arrested at MI, only less than 30% reached the Met II stage, and a considerable fraction (~20%) was abnormal (Fig 2D). These results were consistent with the *in vivo* data (Fig 2A and 2B), thus confirming that *Setdb1* depletion in growing oocytes led to severe defects in meiotic resumption and maturation.

A substantial fraction of *Setdb1* KO oocytes underwent GVBD but failed to progress to the Met II stage *in vivo* and *in vitro* (Fig 2A–2D), suggesting that they were arrested at MI. We therefore assessed whether Setdb1 deficiency affected spindle formation and chromosome dynamics during MI. To this end, GV oocytes were cultured in maturation medium for 5 hours, and the spindle and chromosome structures were examined with α-tubulin and DAPI (4',6-diamidino-2-phenylindole) staining. By the time of examination, the majority of control oocytes that had undergone GVBD were at the metaphase I stage, and a small fraction was at prometaphase I. Most of them exhibited normal spindle and chromosome structures (Fig 2E and 2F). Consistent with the delay in meiotic resumption (Fig 2C), ~50% of *Setdb1* KO oocytes remained arrested at the GV stage, and the ones that had resumed meiosis were mostly at the prometaphase I stage. Nearly 60% of *Setdb1* KO MI oocytes had obvious spindle abnormalities, including dispersed, tread-like, non-polar, and multiple spindles, and ~30% also exhibited defects in chromosome congression or alignment (Fig 2E and 2F). These defects likely played an important part in meiotic arrest at MI. Taken together, our results provided genetic evidence that Setdb1 is critical for meiotic resumption and maturation of mouse oocytes.

### Cdc14b up-regulation contributes to meiotic arrest in *Setdb1* KO oocytes

**Fig 3:**
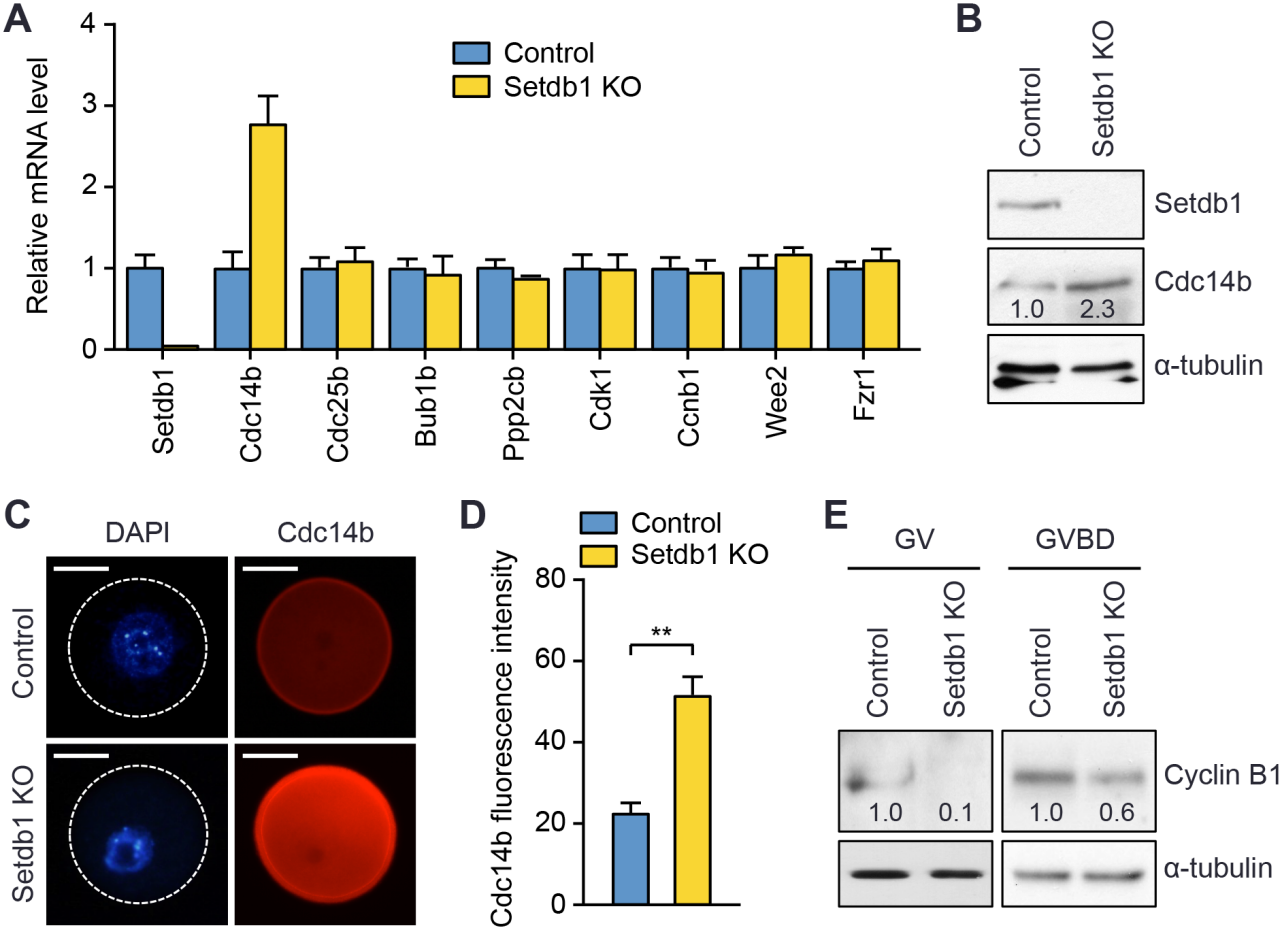
Cdc14b is up-regulated in *Setdb1* KO oocytes. **(A)** qRT-PCR analyses of *Setdb1*, *Cdc14b*, *Cdc25b*, *Bub1b*, *Ppp2cb*, *Cdk1*, *Ccnb1* (encoding cyclin B1), *Wee2*, and *Fzr1* (encoding Cdh1) transcripts in GV oocytes. Shown are relative mRNA levels in control and *Setdb1* KO oocytes (mean ± SEM of triplicate assays). **(B)** Western blot analysis of Setdb1 and Cdc14b in control and *Setdb1* KO GV oocytes, with α-tubulin as a loading control. Each lane contains 100 GV oocytes. Relative band intensities were quantified with the ImageJ software and normalized to the α-tubulin signal. **(C, D)** IF analysis of Cdc14b expression in GV oocytes. **(C)** Representative images of control and *Setdb1* KO oocytes stained with anti-Cdc14b (red) and DAPI (blue). The boundaries of the oocytes are defined by circles. Scale bars, 35 μm. **(D)** Quantification of fluorescence intensity of Cdc14b. Thirty control and 30 *Setdb1* KO oocytes were analyzed, and the data are presented as the mean ± SEM. Statistical comparisons were made using unpaired t-test. **P < 0.01. **(E)** Western blot analysis of Cyclin B1 in GV and GVBD oocytes. GV oocytes were harvested from control and *Setdb1* KO mice. GVBD oocytes were isolated by morphology after culturing GV oocytes in maturation medium for 5 hours. The samples were analyzed by immunoblotting with Cyclin B1 or α-tubulin antibodies. Each lane contains 150 GV oocytes or 85 GVBD oocytes, respectively. Relative band intensities were determined as described above, with the values in control samples being arbitrarily designated as 1.0.

Given the important role of Setdb1 in transcriptional repression, it is likely that the observed defects in meiotic progression were due to aberrant expression of essential genes. Indeed, previous ChIP-Seq analysis in mouse embryonic stem (ES) cells [30] revealed Setdb1 binding, as well as H3K9me3 enrichment, in several genes involved in meiosis, including *Cdc14b*, *Cdc25b*, *Bub1b*, and *Ppp2cb* (S4 Fig). We performed qRT-PCR analysis to compare the expression of these genes, as well as other important meiosis genes *Cdk1*, *Ccnb1* (encoding Cyclin B1), *Wee2*, and *Fzr1* (encoding Cdh1), in *Setdb1* KO and control GV oocytes. The level of *Cdc14b* mRNA was substantially elevated in *Setdb1* KO oocytes (~2.8 fold relative to control), whereas the expression of the other genes tested showed no alterations (Fig 3A). Western blot and IF analyses also confirmed the increase in Cdc14b protein in *Setdb1* KO oocytes (Fig 3B–3D). Notably, the meiotic phenotypes of *Setdb1* KO mice, including meiotic arrest, spindle and chromosome perturbations (Fig 2), were highly similar to the consequences of Cdc14b overexpression [8]. These observations led us to hypothesize that Cdc14b up-regulation may contribute to the meiotic defects associated with *Setdb1* deficiency.

Cdc14b has been shown to negatively regulate meiotic resumption by promoting APC/C-mediated degradation of Cyclin B1 [8]. We assessed whether Cyclin B1 level was altered in *Setdb1* KO oocytes. Western blot analysis revealed that Cyclin B1 was present at low levels in control GV oocytes, but hardly detectable in *Setdb1* KO GV oocytes. Following *in vitro* maturation, both control and KO oocytes that had undergone GVBD exhibited Cyclin B1 accumulation. However, *Setdb1* KO GVBD oocytes had ~40% lower levels of Cyclin B1 compared to their control counterparts (Fig 3E). Thus, Cdc14b up-regulation in *Setdb1* KO oocytes correlated with low levels of Cyclin B1. Indeed, the ability of *Setdb1* KO GV oocytes to undergo GVBD was substantially restored when treated with the proteosome inhibitor MG132 (S5 Fig), consistent with the notion that enhanced Cyclin B1 degradation contributed to the defect in meiotic resumption.

**Fig 4:**
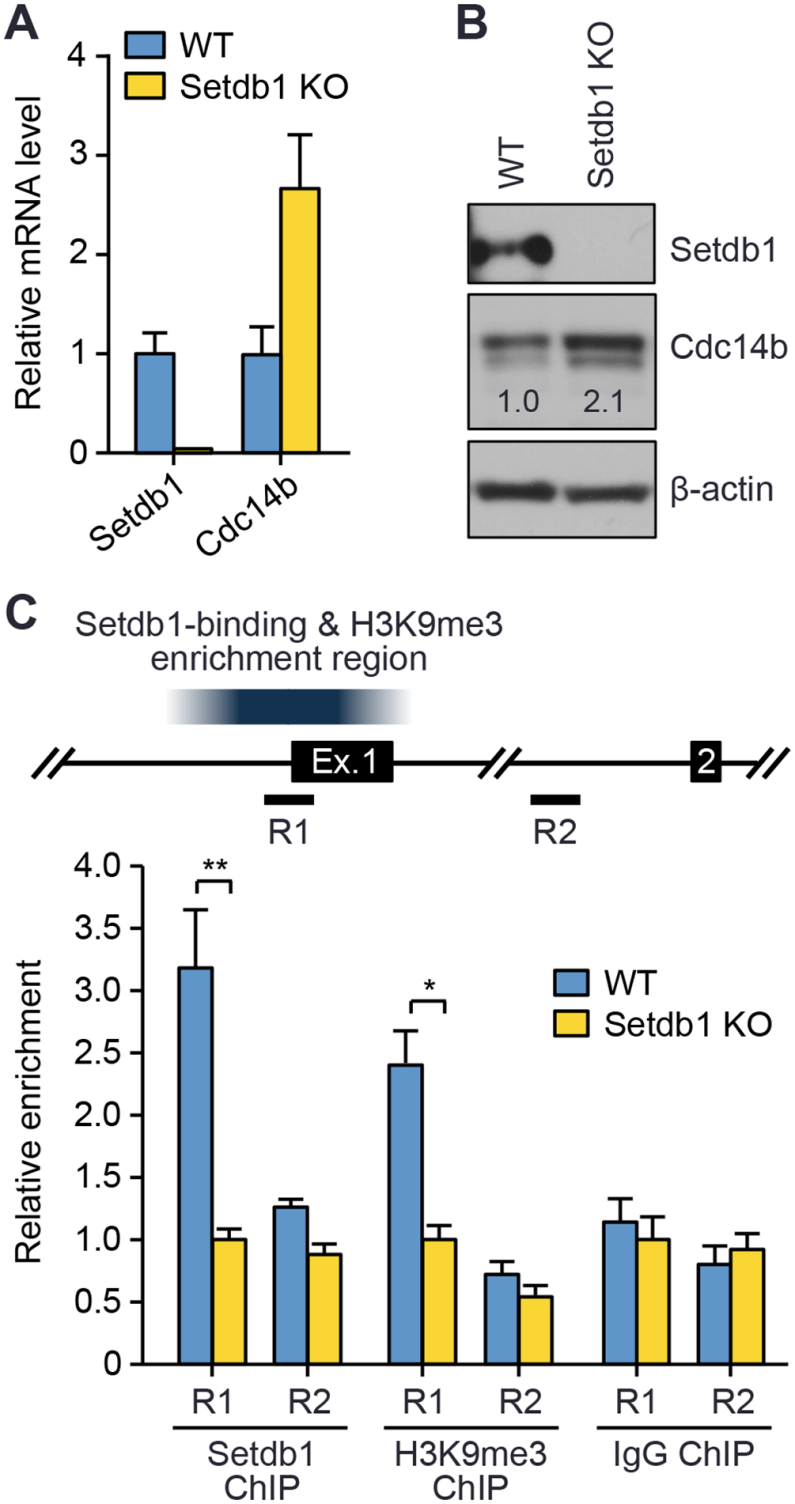
*Setdb1* disruption in mouse ES cells leads to decreased enrichment of H3K9me3 at the *Cdc14b* transcriptional start site and increased *Cdc14b* expression. WT and *Setdb1*^*2lox/1lox*^*/CreERT2* ES cells were treated with 2 μM of 4-hydroxytamoxifen (4-OHT) for 4 days, and the cells (referred to as WT and *Setdb1* KO, respectively, after 4-OHT treatment) were then used for experiments. **(A)** qRT-PCR analysis of *Setdb1* and *Cdc14b* transcripts in WT and *Setdb1* KO ES cells (mean ± SEM of triplicate assays). **(B)** Western blot analysis of Setdb1 and Cdc14b in WT and *Setdb1* KO ES cells, with β-actin as a loading control. Relative band intensities were determined as described in Fig 3. **(C)** ChIP-qPCR analyses. Shown at the top is a portion of the *Cdc14b* locus, including exons 1 and 2 and the proximal promoter, which contains a major Setdb1-binding and H3K9me3 enrichment region (from ChIP-Seq data by Bilodeau et al. [30]). The regions (R1 and R2) analyzed by qPCR are indicated. The data are presented as the relative enrichment of Setdb1, H3K9me3, and control IgG at R1 and R2, with the values of *Setdb1* KO samples being arbitrarily designated as 1.0.

ChIP-Seq analysis of mouse ES cells identified a major Setdb1-binding and H3K9me3 enrichment peak centered at the transcriptional start site (TSS) of *Cdc14b* [30] (S4 Fig), raising the possibility that Setdb1 may directly repress *Cdc14b* transcription by depositing H3K9 methylation marks. We assessed the impact of Setdb1 depletion on H3K9me3 enrichment at the *Cdc14b* locus, as well as *Cdc14b* expression, in ES cells. Because *Setdb1*-null ES cells are not viable [23], we used an inducible approach to deplete Setdb1. *Setdb1*^*2lox/1lox*^ ES cells expressing tamoxifen-inducible Cre (known as Cre-ERT2, a fusion protein consisting of Cre and a mutant form of the estrogen receptor (ERT2) ligand-binding domain) were treated with 4-hydroxytamoxifen (4-OHT), which induces translocation of Cre-ERT2 to the nuclei, resulting in excision of exon 16 of the conditional *Setdb1*^*2lox*^ allele [28]. In agreement with our previous work [28], *Setdb1* mRNA and protein was hardly detectable after 4 days of 4-OHT treatment (Fig 4A and 4B), while the cells (referred to as *Setdb1* KO after treatment) were still viable and looked healthy. In *Setdb1* KO ES cells, both *Cdc14b* mRNA and protein were considerably elevated (Fig 4A and 4B), consistent with the effect of Setdb1 depletion in oocytes (Fig 3A–3D). Chromatin immunoprecipitation coupled to quantitative real-time PCR (ChIP-qPCR) analysis confirmed Setdb1 binding and H3K9me3 enrichment at a region (R1) spanning the *Cdc14b* TSS, but not at a region (R2, negative control) in intron 1 (Fig 4C). Setdb1 depletion led to a significant reduction in H3K9me3 at the R1 region (Fig 4C), indicating that Setdb1 is responsible for H3K9me3 enrichment at the *Cdc14b* TSS. Collectively, these results suggest that *Cdc14b* is a direct transcriptional target of Setdb1.

**Fig 5:**
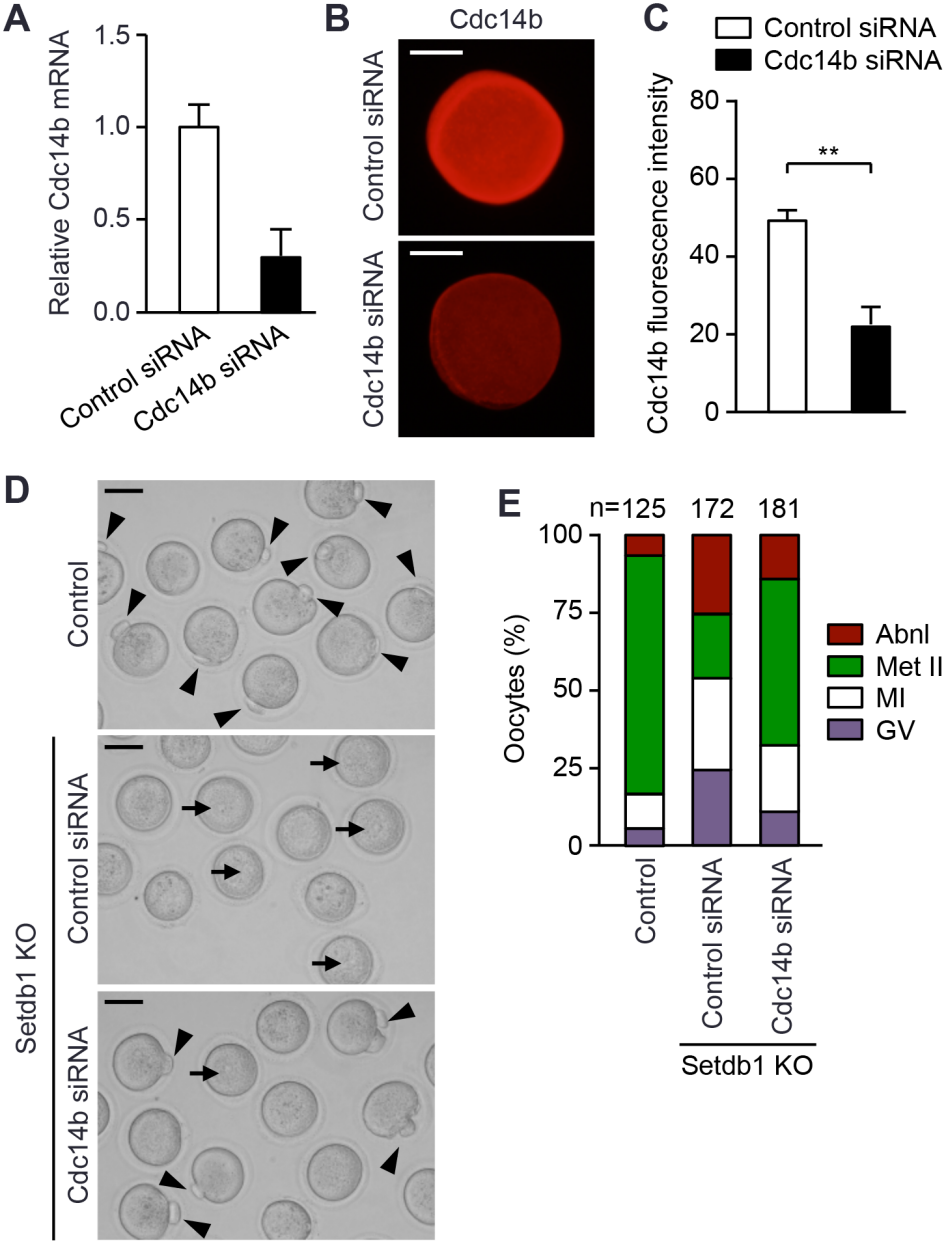
*Cdc14b* depletion in *Setdb1* KO oocytes alleviates meiotic arrest. GV oocytes were harvested from control and *Setdb1* KO mice. *Setdb1* KO oocytes were microinjected with either control siRNA or *Cdc14b* siRNA. These oocytes, as well as control GV oocytes, were incubated in IBMX-containing medium for 24 hours to allow siRNA-mediated *Cdc14b* depletion to occur, followed by *in vitro* maturation in IBMX-free medium for 20 hours. **(A)** qRT-PCR analysis of *Cdc14b* mRNA 24 hours after microinjection. Shown are relative *Cdc14b* mRNA levels in oocytes injected with control siRNA and *Cc14b* siRNA (mean ± SEM of duplicate assays). (**B, C)** IF analysis of Cdc14b 24 hours after microinjection. **(B)** Representative IF images of oocytes injected with control siRNA or *Cdc14b* siRNA. Scale bars, 35 μm. **(C)** Quantification of fluorescence intensity of Cdc14b. Twenty oocytes injected with control siRNA and 20 oocytes injected with *Cdc14b* siRNA were analyzed, and the data are presented as the mean ± SEM. Statistical comparisons were made using unpaired t-test. **P < 0.01. **(D, E)** Determination of meiotic stages after 20 hours of *in vitro* maturation. **(D)** Representative bright-field microscope images of control oocytes and *Setdb1* KO oocytes injected with control siRNA or *Cdc14b* siRNA. Arrowheads and arrows indicate the polar bodies (characteristic of Met II oocytes) and the prominent nucleoli (characteristic of GV oocytes), respectively. Scale bars, 50 μm. **(E)** Percentages of oocytes at different meiotic stages (GV arrested, MI, Met II, and abnormal). In total, 200 KO oocytes were injected with control siRNA and another 200 with Cdc14b siRNA, and 172 and 181 of them, respectively, survived.

To test the hypothesis that excess Cdc14b played a key role in inducing meiotic arrest of *Setdb1* KO oocytes, we assessed the effect of *Cdc14b* depletion on meiotic resumption and maturation. *Setdb1* KO GV oocytes were microinjected with either *Cdc14b* or control small interfering RNA (siRNA), and the injected oocytes, as well as control GV oocytes, were incubated in IBMX-containing medium for 24 hours to allow *Cdc14* depletion to occur while maintaining GV arrest. The oocytes were then released of GV arrest at the same time by washing out IBMX, followed by *in vitro* maturation for 20 hours. Analyses at 24 hours post-injection revealed that *Cdc14b* siRNA led to substantial decreases in *Cdc14b* mRNA (by ~70%) and protein (by ~55%) (Fig 5A–5C). Before the initiation of *in vitro* maturation, almost all oocytes injected with either *Cdc14b* siRNA or control siRNA remained arrested at the GV stage. Following 20 hours of *in vitro* maturation, the majority of *Cdc14b*-depleted oocytes resumed meiosis, with over 50% reaching the Met II stage, whereas microinjection of control siRNA had no effect on the meiotic arrest phenotype (Fig 5D and 5E, compare with Fig 2D). The improvement in meiotic progression appeared to be partially due to the amelioration of spindle defects during MI, as examination of *Cdc14b*-depeleted oocytes after 6 hours of *in vitro* maturation revealed a significant lower proportion of MI oocytes exhibiting abnormal spindles (S6 Fig). Taken together, up-regulation of Cdc14b, to a large extent, contributed to the defects in meiotic resumption and maturation in *Setdb1* KO oocytes.

### *Setdb1* KO oocytes show derepression of retrotransposons and DNA damage

**Fig 6:**
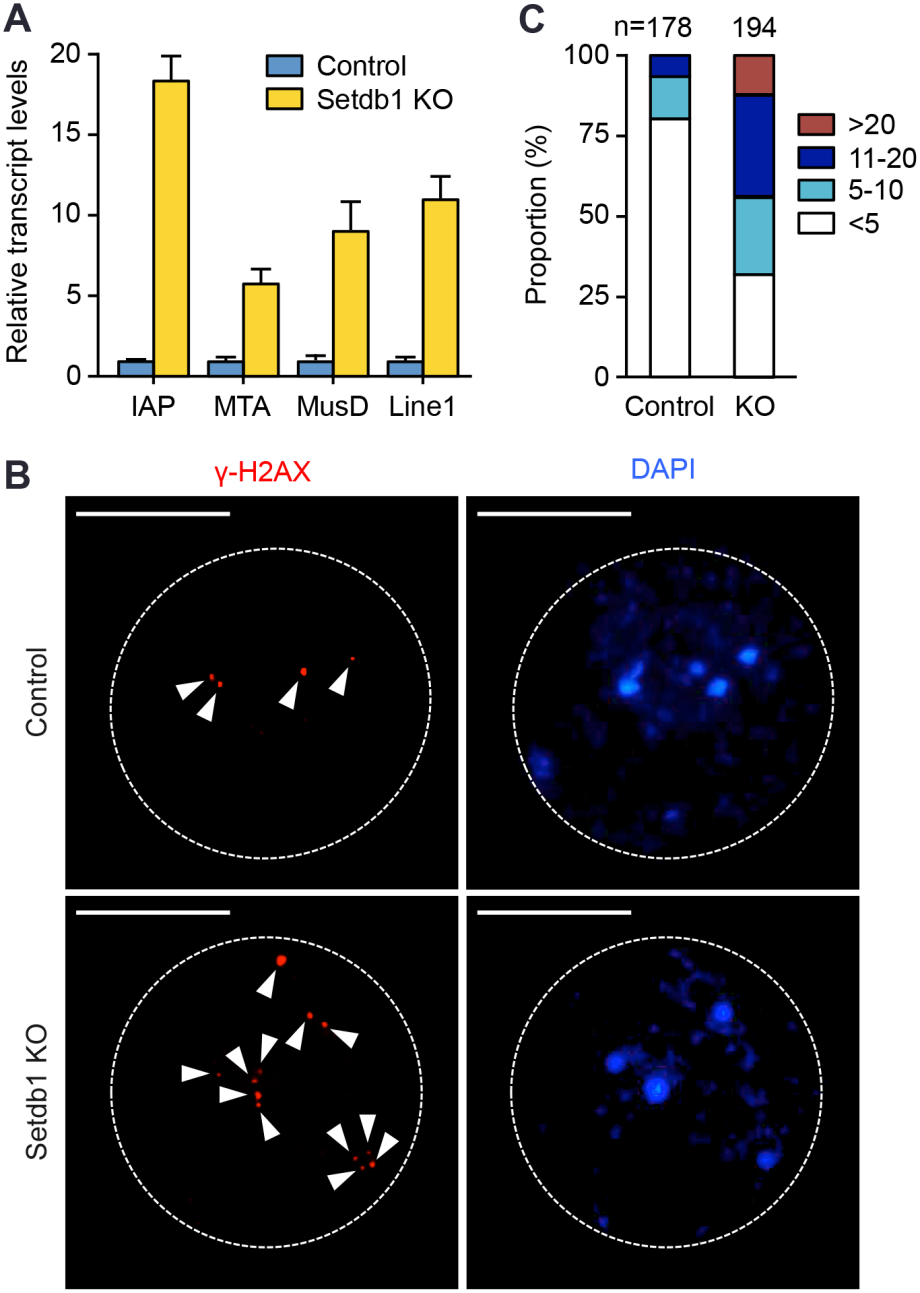
*Setdb1* KO oocytes show derepression of retrotransposons and increased DNA double-strand breaks. **(A)** qRT-PCR analysis of retrotransposon transcripts in control and *Setdb1* KO GV oocytes. The data are presented as the mean ± SEM of triplicate assays. *IAP*, intracisternal A particles; *Line1*, long interspersed nuclear element 1; *MTA*, mouse transposon A; *MusD*, the type D murine LTR retrotransposon. **(B, C)** GV oocytes were examined for DNA double-strand breaks (DSBs) with anti-γ-H2AX staining. **(B)** Representative γ-H2AX (red) and DAPI (blue) images of control and *Setdb1* KO oocytes. The nuclei of oocytes are circled, and the γ-H2AX foci are indicated by arrowheads. Scale bars: 10 μm. **(C)** The proportions of oocytes with various numbers of γ-H2AX foci in the nuclei. In total, 178 control oocytes and 194 KO oocytes were examined.

A subset of retrotransposons, including long terminal repeat (LTR)-containing endogenous retroviruses (ERVs) and non-LTR long interspersed nuclear element 1 (Line1), maintain the ability to retrotranspose and thus need to be actively suppressed [19]. Recent studies have demonstrated that Setdb1 is essential for retrotransposon silencing in undifferentiated ES cells, early embryos, and PGCs [17,18]. To determine whether Setdb1 is also required to silence retrotransposons in growing oocytes, we measured the transcript levels of retrotransposons in *Setdb1* KO and control GV oocytes. As shown in Fig 6A, *Setdb1* depletion led to marked up-regulation of *Line1* and several ERV elements, including intracisternal A particles (*IAP*), mouse transposon A (*MTA*), and the type D murine LTR retrotransposon (*MusD*).

Derepression of retrotransposons could lead to genomic instability [31]. Changes in global H3K9me2 levels could also impair chromatin structure and genome stability [24]. We measured DNA double-strand breaks (DSBs) by phosphorylated H2AX (γ-H2AX) immunostaining and found that *Setdb1* KO oocytes had substantially more γ-H2AX foci than control oocytes (Fig 6B and 6C). DNA DSBs have been shown to adversely affect oocyte meiotic progression [32,33]. It is thus likely that DNA damage induced by *Setdb1* depletion also played a role in the meiotic arrest phenotype.

### Embryos derived from *Setdb1* KO oocytes exhibit progressive developmental delays and fail to reach the blastocyst stage

**Fig 7:**
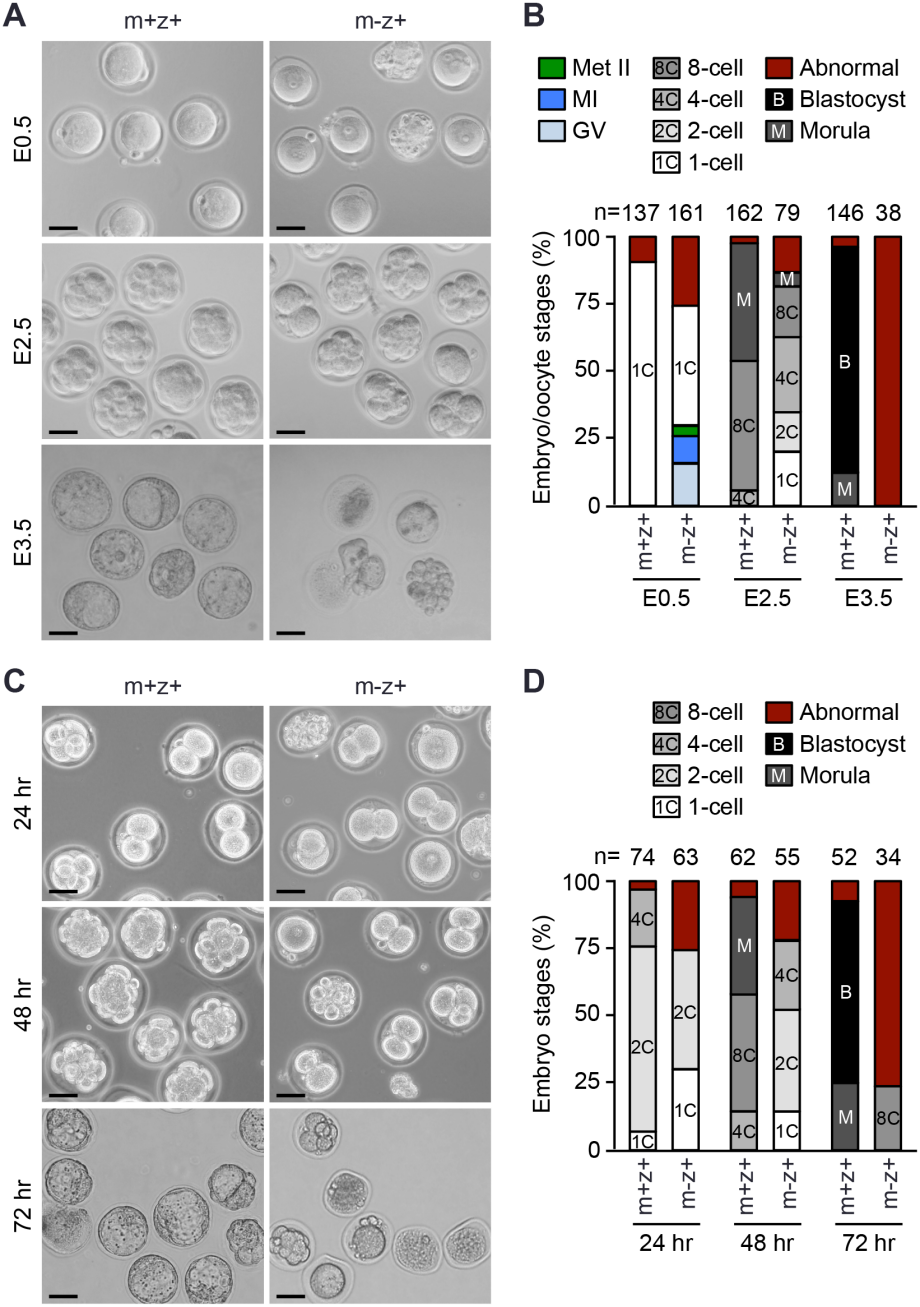
Embryos lacking maternal Setdb1 exhibit progressive developmental delays and fail to develop to blastocysts. **(A, B)** Superovulated control and *Setdb1* KO females were mated with WT males, and embryos (as well as unfertilized GV, MI, and Met II oocytes) were collected at E0.5, E2.5, and E3.5, respectively, and their developmental stages determined by morphologies. Shown are representative images **(A)** and percentages of embryos, as well as unfertilized oocytes, at different stages **(B)**. Abnormal embryos/oocytes included those exhibiting abnormal morphologies and undergoing degeneration. The total numbers of embryos/oocytes examined for each genotype at each time point are indicated. **(C, D)**. Embryo culture *in vitro*. Superovulated control and *Setdb1* KO females were mated with WT males, and morphologically normal *Setdb1*^*m+z+*^ and *Setdb1*^*m-z+*^ zygotes were isolated at E0.5. The embryos were cultured for 24, 48, and 72 hours *in vitro*, and their developmental stages determined by morphologies. Shown are representative images **(C)** and percentages of embryos at different stages **(D)**. The total numbers of embryos examined for each genotype at each time point are indicated.

Despite the defects in meiotic resumption and maturation, a considerable fraction of *Setdb1* KO oocytes was able to develop to the Met II stage (Fig 2A, 2B and 2D). To assess the fertilizability and developmental competence of these oocytes, superovulated *Setdb1* KO females were mated with WT males, and embryos (referred to as *Setdb1*^*m-z+*^ for maternal deficient and zygotic wild-type) were collected at various time points. Examination of the embryos/oocytes collected at 0.5 dpc (E0.5) suggested that most *Setdb1* KO Met II oocytes were fertilizable, as the number of zygotes (~44%) recovered (Fig 7A and 7B) was similar to that of Met II oocytes collected from the oviducts of superovulated *Setdb1* KO mice (Fig 2B), and only a small number of unfertilized Met II oocytes (~4%) were observed (Fig 7B). Consistent with the meiotic arrest phenotype, considerable numbers of GV (~16%), MI (~10%), and abnormal (~26%) oocytes were also present at E0.5 (Fig 7A and 7B). At E2.5, the vast majority of control embryos (referred to as *Setdb1*^*m+z+*^) were at the 8-cell and morula stages (~48% and ~44%, respectively). In contrast, considerable fractions of *Setdb1*^*m-z+*^ embryos remained at the 1-cell (~20%), 2-cell (~15%) and 4-cell (~28%) stages, and only ~19% developed to the 8-cell stage and very few (~5%) appeared to be morulae with abnormal morphologies (Fig 7A and 7B). At E3.5, *Setdb1*^*m+z+*^ embryos were predominantly at the blastocyst stage (~84%), as expected, whereas the small numbers of *Setdb1*^*m-z+*^ embryos recovered (3.2 per litter on average) were all undergoing degeneration (Fig 7A and 7B). These data revealed that, although *Setdb1*-depleted Met II oocytes were fertilizable, embryos lacking maternal Setdb1 exhibited progressive delays in development, with most of them undergoing degeneration prior to the morula stage and none of them reaching the blastocyst stage.

To confirm the embryonic phenotype, we isolated morphologically “normal” *Setdb1*^*m+z+*^ and *Setdb1*^*m-z+*^ zygotes at E0.5 and cultured them for 24–72 hours *in vitro*. As shown in Fig 7C and 7D, the results were generally consistent with the *in vivo* data (Fig 7A and 7B), thus strengthening the conclusion that the development of *Setdb1*^*m-z+*^ embryos was severely delayed and defective.

### *Setdb1*^*m-z+*^ zygotes display impaired mitotic cell cycle progression

**Fig 8:**
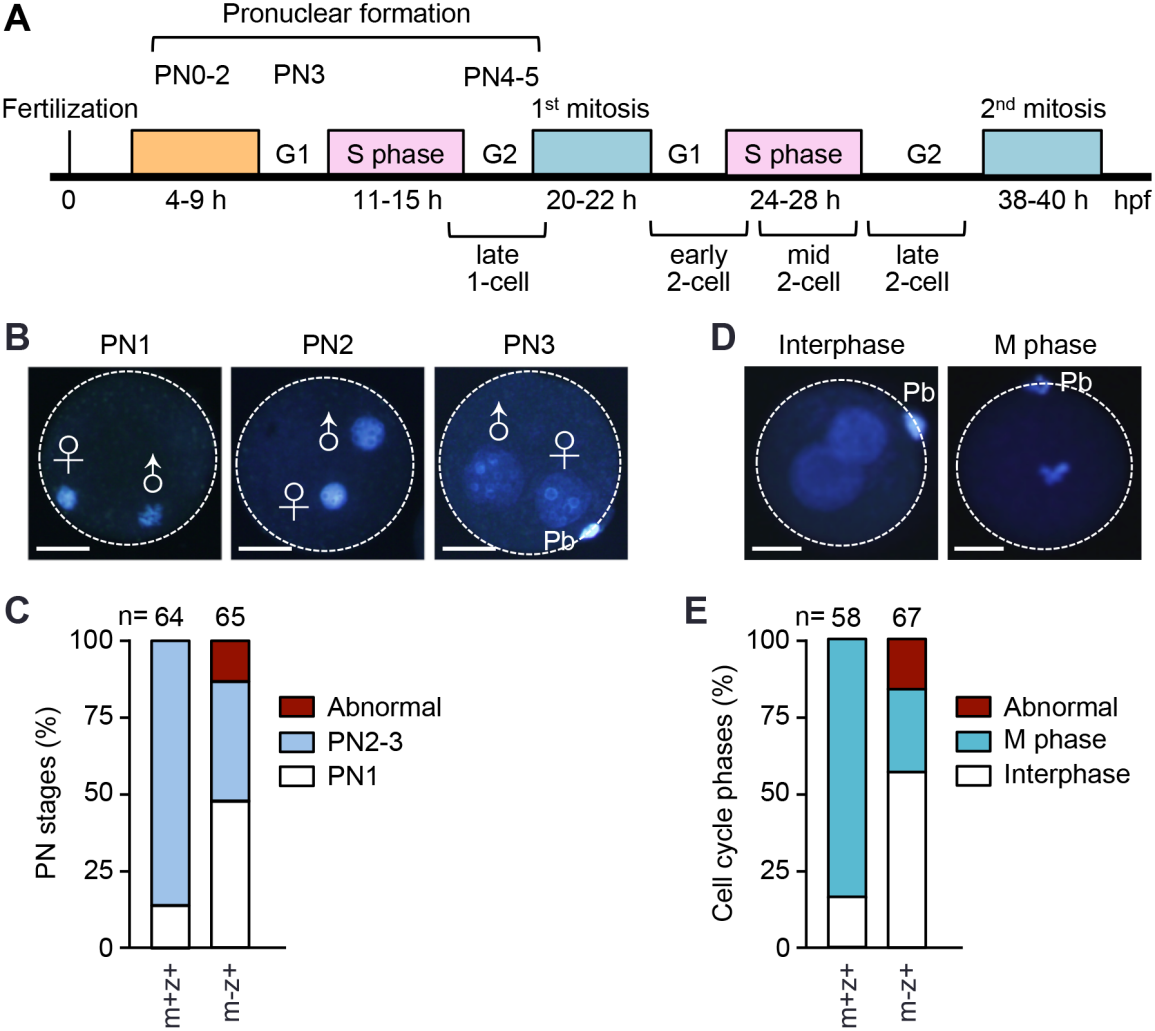
*Setdb1*^*m-z+*^ zygotes show severe delays in pronuclear maturation and entry into the first mitosis. **(A)** Schematic representation of the timing of cell cycle phases of 1-cell and 2-cell stage embryos. (**B, C**) Determination of pronuclear (PN) stages after *in vitro* fertilization. Met II oocytes from *Setdb1* KO and control mice were fertilized *in vitro* with sperm from WT mice, and at 5 hours post-fertilization (hpf), the zygotes (*Setdb1*^*m-z+*^ and *Setdb1*^*m+z+*^, respectively) were stained with DAPI (blue) to determine their PN stages. **(B)** Representative zygotes at PN1, PN2, and PN3 stages. The boundaries of the zygotes are defined by circles, and the male and female pronuclei are indicated. Pb, polar body. Scale bars, 30 μm. **(C)** The percentages of PN1, PN2-3, and abnormal zygotes. The total numbers of embryos examined are indicated. (**D, E**) *Setdb1* KO and control females were mated with WT males, zygotes (*Setdb1*^*m-z+*^ and *Setdb1*^*m+z+*^, respectively) collected at E0.5 were incubated in the presence of colcemid for 18 hours and then stained with DAPI to determine the cell cycle stages. **(D)** Representative zygotes at interphase and M phase. The boundaries of the zygotes are defined by circles. Pb, polar body. Scale bars, 30 μm. **(E)** The percentages of interphase, M phase, and abnormal zygotes. The total numbers of embryos examined are indicated.

The male and female pronuclei form shortly after fertilization and then expand and migrate toward each other before the first mitosis (Fig 8A). In examining E0.5 embryos, we noticed that the pronuclear (PN) stages of *Setdb1*^*m-z+*^ zygotes were frequently less advanced, as compared to *Setdb1*^*m+z+*^ zygotes. To exclude the possibility that the delayed PN maturation displayed by *Setdb1*^*m-z+*^ zygotes were due to different timing of fertilization, we carried out *in vitro* fertilization experiments. Met II oocytes from *Setdb1* KO and control mice were fertilized with WT sperm, and the PN stages were determined by DAPI staining at 5 hours post-fertilization (hpf). *Setdb1*^*m-z+*^ zygotes were generally delayed in PN maturation. Whereas the vast majority (~85%) of *Setdb1*^*m+z+*^ embryos reached the PN2-3 stages at 5 hpf, only less than 40% of *Setdb1*^*m-z+*^ embryos did, and the rest was mostly at the PN1 stage (Fig 8B and 8C).

Delayed PN maturation could reflect impaired cell cycle progression. We therefore measured M-phase entry of zygotes. Control and *Setdb1* KO females were mated with WT males, and zygotes collected at E0.5 were cultured for 18 hours in the presence of colcemid, which depolymerizes microtubules and arrests zygotes at mitosis. Most *Setdb1*^*m+z+*^ zygotes (~85%) arrested at the M phase with mitotic condensed chromosomes. In contrast, a much smaller fraction (~27%) of *Setdb1*^*m-z+*^ zygotes reached the M phase, and the majority (nearly 60%) remained in the interphase (Fig 8D and 8E). Collectively, these results indicated that *Setdb1*^*m-z+*^ embryos had severe defects in progressing through the first mitotic cell cycle. It is likely that subsequent cell cycles were also impaired, given the progressive developmental delays exhibited by these embryos.

### Restoration of Setdb1 activity in *Setdb1* KO GV oocytes partially rescues the meiotic and embryonic defects

**Fig 9:**
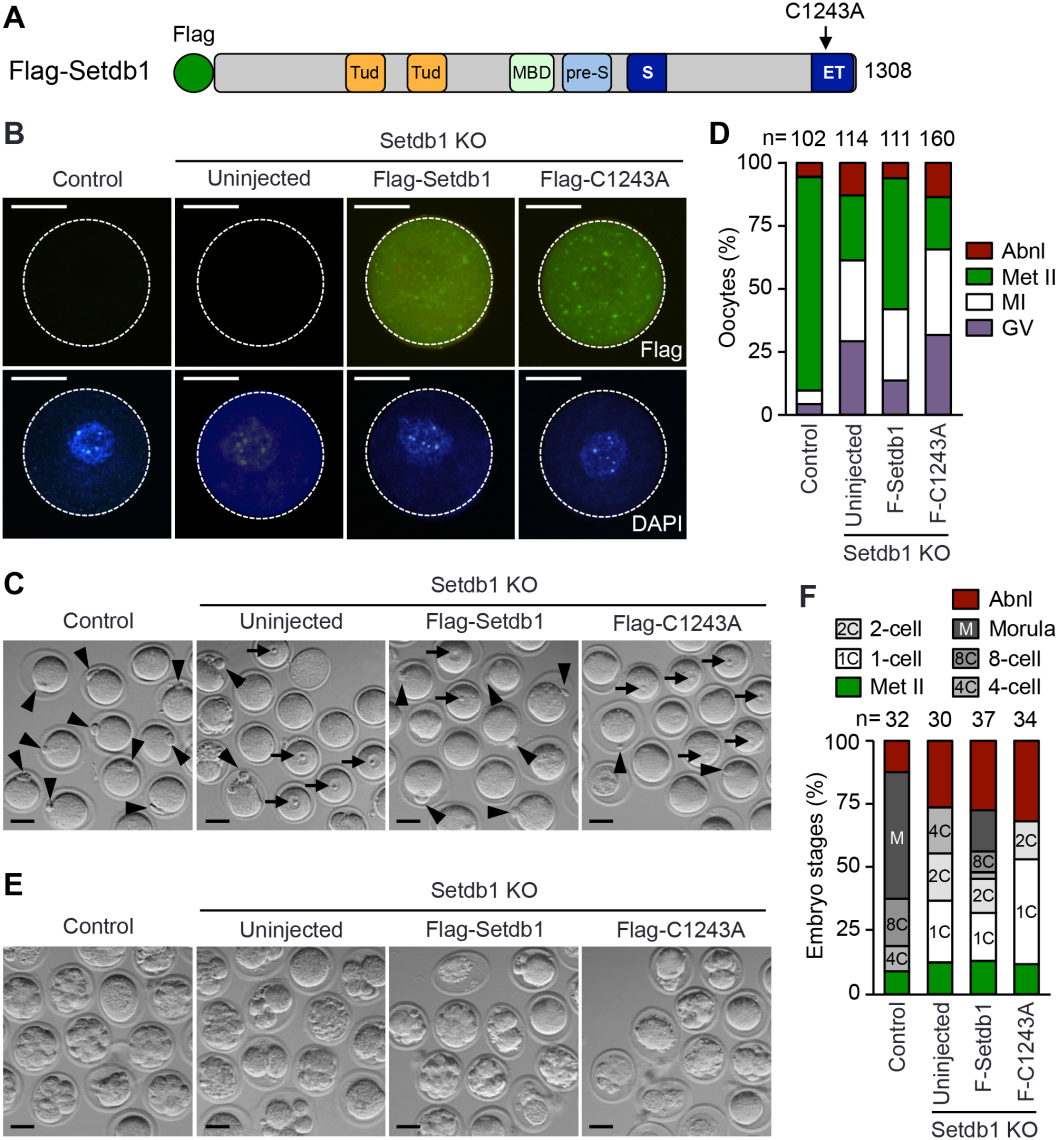
Expression of WT, but not inactive, Setdb1 in Setdb1 KO GV oocytes partially rescues the meiotic and embryonic defects. **(A)** Structure of the Setdb1 protein showing the major functional domains. Tud and Tud, tandem Tudor domain; MBD, methyl-CpG-binding domain; pre-S, pre-SET domain; S and ET, bifurcated SET domain. The location of the point mutation altering cysteine 1243 to alanine (C1243A) is indicated. The WT and mutant Setdb1 proteins expressed in oocytes have an N-terminal Flag tag. **(B-D)** Fully-grown GV oocytes were harvested from control and *Setdb1* KO mice. *Setdb1* KO oocytes were injected with mRNAs encoding Flag-tagged WT Setdb1 (Flag-Setdb1) or catalytically inactive Setdb1 (Flag-C1243A). The injected oocytes, as well as uninjected control and *Setdb1* KO oocytes, were incubated in IBMX-containing medium for 2 hours to allow the expression of Flag-tagged Setdb1 proteins, followed by *in vitro* maturation in IBMX-free medium for 18 hours. **(B)** Representative IF images showing expression of Flag-Setdb1 proteins (green) 2 hours after mRNA injection. The nuclei were stained with DAPI (blue). The boundaries of the oocytes are defined by circles. Scale bars, 35 μm. **(C)** Representative bright-field microscope images of uninjected oocytes and oocytes expressing Flag-Setdb1 or Flag-C1243A following 18 hours of *in vitro* maturation. Arrowheads and arrows indicate the polar bodies (characteristic of Met II oocytes) and the prominent nucleoli (characteristic of GV oocytes), respectively. Scale bars, 50 μm. **(D)** Percentages of oocytes at different meiotic stages (GV arrested, MI, Met II, and abnormal) following 18 hours of *in vitro* maturation. The total numbers of oocytes examined are indicated. **(E, F)** Met II oocytes derived from *in vitro* maturation (described above) were inseminated with sperm from WT mice and then cultured for 48 hours. **(E)** Representative images of embryos derived from the indicated Met II oocytes. Scale bars, 50 μm. **(F)** Percentages of different stages of preimplantation embryos, as well unfertilized oocytes (Met II), are shown. The total numbers of embryos/oocytes examined are indicated.

To determine whether the meiotic and embryonic phenotypes can be rescued by Setdb1 re-expression and whether its catalytic activity is required, *Setdb1* KO GV oocytes were microinjected with mRNA encoding Flag-tagged WT Setdb1 (Flag-Setdb1) or Setdb1 with a point mutation altering cysteine 1243 to alanine (Flag-C1243A). The C1243A mutation is located in the bifurcated SET domain (Fig 9A) and abolishes the catalytic activity [14,15]. The mRNAs for microinjection were produced by *in vitro* transcription (with Poly(A) tailing) using plasmid constructs as templates (S7 Fig). IF analysis, performed 2 hours post-injection, confirmed the expression of Flag-tagged Setdb1 proteins (Fig 9B). Following 18 hours of *in vitro* maturation, the vast majority of *Setdb1* KO oocytes expressing Flag-Setdb1 resumed meiosis, with over 50% reaching the Met II stage, albeit the meiotic defects were not completely prevented, as compared to control oocytes. In contrast, the expression of inactive Setdb1 (Flag-C1243A) had no effect on the meiotic arrest phenotype, with ~30% of oocytes remaining arrested at the GV stage and only ~20% reaching the Met II stage, similar to uninjected *Setdb1* KO oocytes (Fig 9C and 9D).

To assess the effect of Setdb1 re-expression on embryonic defects, the Met II oocytes obtained from the *in vitro* maturation experiments (described above) were inseminated with WT sperm, and the embryos were cultured for 48 hours. In all four groups, the majority of oocytes were fertilized, although small fractions (~10%) of unfertilized Met II oocytes were observed. After 48 hours of *in vitro* development, most embryos derived from control oocytes developed to the morula (~55%) or 8-cell (~20%) stages (Fig 9E and 9F), similar to the results from *in vitro* development of *Setdb1*^*m+z+*^ embryos (Fig 7C and 7D). Among the embryos derived from *Setdb1* KO oocytes, injected or uninjected, considerable fractions (~30–35%) were morphologically abnormal or undergoing degeneration, indicating that the developmental competence of mature eggs derived from Setdb1-expressing oocytes were still severely compromised. However, in the Flag-Setdb1 group, nearly 20% of the embryos reached the morula stage, and another ~10% developed to the 8-cell stage, albeit substantial fractions were arrested at the 1-cell (22%) or 2-cell (16%) stages. In contrast, in the uninjected group, none of the embryos developed beyond the 4-cell stage, and in the Flag-C1243A group, ~50% of embryos were arrested at the 1-cell stage and only a small fraction reached the 2-cell stage (Fig 9E and 9F). It is also noteworthy that *Setdb1*^*m-z+*^ zygotes derived from natural mating failed to develop beyond the 8-cell stage *in vitro*, even with 72 hours of culture (Fig 7C and 7D). Thus, restoration of Setdb1 activity in *Setdb1* KO GV oocytes not only facilitated meiotic progression but also improved the ability of mature oocytes to support early embryogenesis. The partial effects on meiotic and embryonic phenotypes could be because the Setdb1 levels were not optimal or some genomic/chromatin defects that had already occurred could not be remedied by Setdb1 re-expression.

## Discussion

**Fig 10:**
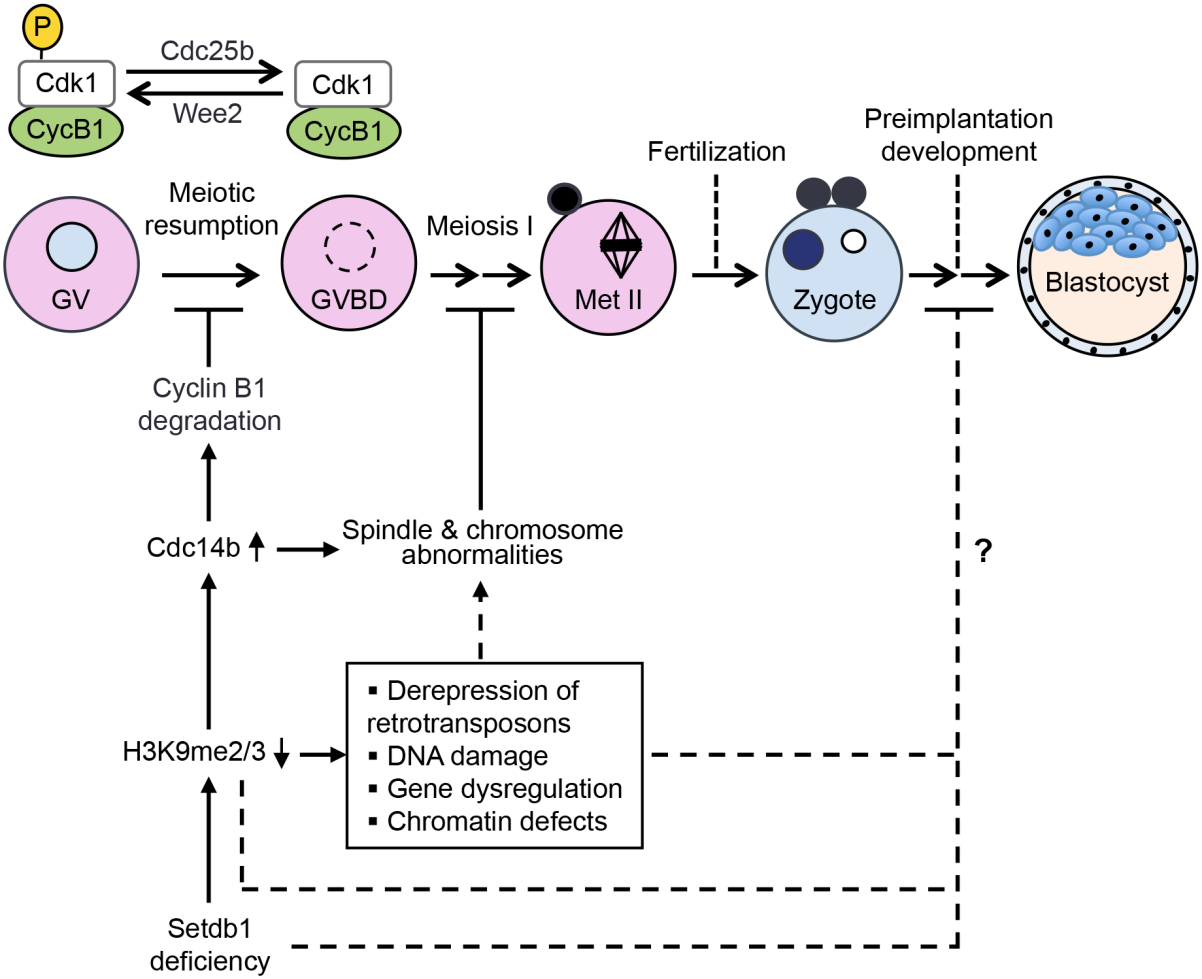
Proposed effects of Setdb1 depletion on meiotic progression and preimplantation development. Setdb1 depletion in growing oocytes leads to decreases in H3K9-methyl marks. One consequence is elevated expression of Cdc14b, which leads to Cyclin B1 degradation and inhibition of meiotic resumption. Cdc14b upregulation also contributes to subsequent spindle and chromosomal abnormalities and meiotic arrest at meiosis I. Other consequences of decreases in H3K9 methylation, including derepression of retrotransposons, DNA damage, altered gene expression, and chromatin defects, likely also play important roles in inducing spindle and chromosome defects at meiosis I. The mechanisms by which Setdb1 depletion leads to preimplantation development defects remain to be determined. The consequences of Setdb1 depletion (e.g. H3K9 methylation decreases, altered gene expression, and genomic and chromosomal instability) and/or the lack of maternal Setdb1 itself may affect essential cellular processes during preimplantation development.

In summary, we demonstrated that, in mouse, maternal Setdb1 controls global H3K9me2 level in developing oocytes, plays crucial roles in meiotic progression, and is essential for preimplantation development. Conditional deletion of *Setdb1* in growing oocytes resulted in inhibition of meiotic resumption and impairment of meiotic progression following GVBD, largely due to up-regulation of Cdc14b, a negative regulator of meiotic progression. Other consequences of Setdb1 depletion and altered H3K9 methylation, including derepression of retrotransposons, increased DNA damage, aberrant expression of additional genes, and chromatin defects, likely also contributed to the meiotic arrest phenotype (Fig 10). Although some *Setdb1*-deficient oocytes developed to fertilizable eggs, embryos derived from these eggs were severely defective in cell cycle progression and failed to reach the blastocyst stage. Importantly, re-expression of WT Setdb1, but not catalytically inactive Setdb1, in *Setdb1* KO GV oocytes partially rescued the meiotic and embryonic defects, suggesting that the catalytic activity of maternal Setdb1 is essential for meiotic progression and early embryogenesis. Nevertheless, further work is required to determine how depletion of maternal Setdb1 leads to severe defects in preimplantation development. The consequences of Setdb1 deficiency, including decreased H3K9 methylation, altered gene expression, and genomic and chromatin defects, and/or the lack of maternal Setdb1 itself may affect essential cellular processes (Fig 10). Our work demonstrates that *Setdb1* is a maternal-effect gene essential for fertility.

Meiotic progression is accompanied by epigenetic changes. However, little is known about the functional significance of these changes, as well as the key epigenetic regulators involved. The finding that Setdb1, the predominant histone H3K9 KMT in oocytes, regulates the expression of Cdc14b, a phosphatase that counteracts Cdk1 activity [7], uncovers a functional link between the epigenetic machinery and the major signaling pathway that governs meiotic progression. While the roles of Cdc14b, as well as the mechanisms involved, in meiosis are not fully understood, there is evidence that Cdc14b promotes Cyclin B1 degradation and regulates meiotic spindle dynamics [8]. *Setdb1*-deficient oocytes had elevated Cdc14b levels, which correlated with Cyclin B1 reduction, meiotic arrest in GV and MI stages, and abnormal meiotic spindles. Importantly, siRNA-mediated knockdown of *Cdc14b* in *Setdb1*-deficient oocytes considerably alleviated the defects in meiotic resumption and maturation. These findings led us to conclude that excess Cdc14b was largely responsible for the meiotic defects in *Setdb1*-deficient oocytes. Our results suggest that Setdb1, by keeping Cdc14b below a threshold level, plays important roles in controlling the timing of meiotic resumption and in regulating spindle formation and function following GVBD. The limited number of oocytes one can obtain makes it difficult to determine whether Setdb1 directly or indirectly regulates *Cdc14b* expression. We performed ChIP analysis in mouse ES cells instead, because Setdb1 depletion leads to identical effect on *Cdc14b* expression in oocytes and ES cells. Our results confirmed that Setdb1 binds to and deposits the repressive H3K9me3 mark at a region spanning the *Cdc14b* TSS, thus strongly suggesting that Setdb1 directly represses *Cdc14b* transcription.

Because a subset of retrotransposons remains active, regulatory mechanisms have evolved to suppress their expression. DNA methylation and Setdb1-mediated H3K9 methylation have been shown to silence retrotransposons in somatic cells and undifferentiated cells (ES cells, early embryos, and PGCs), respectively [17,18,19]. Our finding that Setdb1 is also required for suppressing retrotransposons in developing oocytes suggests a more general role for Setdb1-mediated H3K9 methylation in retrotransposon silencing. Derepression of retrotransposons is often detrimental to the genome. Indeed, *Setdb1*-deficient oocytes showed increased DNA damage, which likely contributed to the meiotic and embryonic defects.

Due to meiotic arrest, *Setdb1* KO female mice produced considerably fewer Met II oocytes, which were mostly fertilizable. However, embryos lacking maternal Setdb1 (*Setdb1*^*m-z+*^) exhibited progressive developmental delays, with the vast majority being degenerated prior to the morula stage and none reaching the blastocyst stage. The significantly more severe phenotype and earlier lethality of *Setdb1*^*m-z+*^ embryos, compared to embryos deficient for zygotic Setdb1 (*Setdb1*^*-/-*^ embryos develop to the blastocyst stage and die at 3.5–5.5 dpc) [23], suggest that preimplantation development mainly (if not entirely) relies on maternal Setdb1.

We showed that the progression of the first mitotic cell cycle was severely impaired in *Setdb1*^*m-z+*^ embryos, suggesting that maternal Setdb1 may be important for the transitioning from meiotic to mitotic divisions. Based on the progressive developmental delays of these embryos, it is highly likely that subsequent cleavage divisions were also impaired. While the molecular mechanisms underlying these defects remain to be determined, our results suggest that the catalytic activity of maternal Setdb1 is essential for early embryogenesis. Because many cell cycle regulators regulate both meiosis and mitosis [5], one possibility is that misregulated genes in *Setdb1*-deficient oocytes not only led to meiotic arrest, but also contributed to the defects in mitotic cell cycle progression in early embryos. Previous studies have shown that Cdc14b is a component of the G2 checkpoint that prevents entry into mitosis following DNA damage and that Cdc14b overexpression in zygotes causes mitotic arrest at the 1- and 2-cell stages and inhibits ZGA [9,34]. However, the phenotype of *Setdb1*^*m-z+*^ embryos was much less severe, as compared to Cdc14b-overexpressing embryos [9]. In mouse, ZGA occurs at late two-cell stage and is essential for further development. The fact that a significant fraction of *Setdb1*^*m-z+*^ embryos developed to the 4-cell stage and beyond argues against a general failure in ZGA as the major cause of preimplantation development defects. It is therefore unlikely that Cdc14b elevation played a major role in the developmental defects of *Setdb1*^*m-z+*^ embryos. It is also possible that Setdb1-deficient mature oocytes, albeit fertilizable, had chromatin and genomic defects that impair cellular processes in preimplantation embryos. Maternal Setdb1 persists through preimplantation development and exhibits dynamic localization patterns, implying multiple roles during early embryogenesis [35]. Thus, another possibility is that the lack of Setdb1 activity in early embryos, rather than changes in gene expression and chromatin in oocytes, was mainly responsible for the embryonic defects. These possibilities are not mutually exclusive, and they may all have contributed to the phenotypic abnormalities.

## Methods

### Mice

Experimental mice were maintained on a C57BL/6-129Sv hybrid background and used in accordance with the National Institutes of Health Guide for the Care and Use of Laboratory animals, with Institutional Care and Use Committee-approved protocols at The University of Texas MD Anderson Cancer Center (MDACC). The *Setdb1*^*3lox*^, *Setdb1*^*2lox*^ (conditional), and *Setdb1*^*1lox*^ (null) alleles (schematically shown in S1 Fig) were described previously [28]. *Zp3-Cre* transgenic mice were used to disrupt *Setdb1* in growing oocytes (the breeding scheme is shown in S1 Fig). Mice were genotyped by PCR, and the primers used are listed in S1 Table.

### *LacZ* staining

Ovaries from 4-week-old *Setdb1*^*3lox/+*^ and WT mice were fixed in 2% paraformaldehyde-0.1% glutaraldehyde in phosphate buffered saline (PBS) for 1 hour on ice and permeabilized in Rinse buffer (2mM MgCl_2_, 0.01% sodium deoxycholate, 0.02% NP-40 in PBS) three times (30 min each) at room temperature. The tissues were then incubated in X-gal solution (1mg/ml X-gal, 5mM potassium ferricyanide and 5mM potassium ferrocyanide in Rinse buffer) overnight at 37°C, post-fixed in 10% formalin at room temperature, and embedded in paraffin using standard protocols. Ovary sections were deparaffinized and counterstained with nuclear fast red (Sigma) and mounted.

### Oocyte collection and *in vitro* maturation

Fully-grown GV oocytes were obtained from the ovaries of 4–6 week-old female mice 48 hours after intra-peritoneal injection of 5 IU of pregnant mare’s serum gonadotrophin (PMSG, Sigma). Ovaries were placed in a Petri dish with pre-warmed (37°C) M2 medium (Invitrogen) supplemented with 200 μM of 3-isobutyl-1-methylxanthine (IBMX, Sigma) so as to prevent oocytes from undergoing GVBD. GV oocytes were released by puncturing antral follicles with a fine needle on the stage of a dissecting microscope. To obtain Met II oocytes, 5 IU of human chorionic gonadotrophin (hCG, Sigma) was administered 48 hours after PMSG injection. Mice were euthanized the following morning, and oocytes were collected from the oviducts and released into a hyaluronidase/M2 solution for removal of the cumulus cells. For *in vitro* maturation, oocytes were washed and cultured in IBMX-free M16 medium (Millipore) for various periods of time at 37°C in 5% CO_2_ atmosphere.

### *In vitro* fertilization

Epididymis was dissected into pre-warmed (37°C) Human Tubal Fluid (HTF). 4 μl of fresh sperm were added to a 200 μl HTF drop covered with mineral oil and capacitated for 2 hours in the incubator. Then Met II oocytes, either obtained from superovulated mice or derived from *in vitro* maturation, were added directly to the sperm suspension. After incubating for a maximum of 5 hours at 37°C, 5% CO_2_ in HTF, eggs were washed with KSOM medium and incubated for various periods of time in KSOM medium at 37°C in 5% CO_2_ atmosphere.

### Embryo collection and *in vitro* development

*Setdb1* KO and control mice were superovulated and mated with WT males. Fertilized oocytes (zygotes) were collected from the oviducts at E0.5 and released into a hyaluronidase/M2 solution for dissociation. E2.5 embryos were flushed out the infundibulum of the oviducts, and E3.5 embryos were flushed out of the uterus. For *in vitro* embryo development, zygotes were cultured in KSOM medium at 37°C in 5% CO_2_ atmosphere for 24–72 hours.

### Gene knockdown and ectopic expression in GV oocytes

To knockdown *Cdc14b* or express Flag-tagged Setdb1 proteins in *Setdb1* KO GV oocytes, siRNAs or mRNAs were introduced by microinjection. Briefly, fully-grown GV oocytes were isolated 48 hours after PMSG injection, kept in M2 medium containing IBMX (200 μM), and injected with either 4 μM of siRNA (*Cdc14b* or control) or 10 pl of mRNA (*Flag-Setdb1* or *Flag-C1243A*, 0.2 μg/μl) with a FemtoJet microinjector. The injected oocytes were incubated in IBMX-containing medium either for 24 hours after siRNA injection or for 2 hours after mRNA injection, followed by *in vitro* maturation in IBMX-free medium. The Silencer Select *Cdc14b* siRNA (s104254) and Silencer Select Negative Control No. 1 siRNA (4390843) were purchased from Life Technologies. ARCA (Anti-Reserve Cap Anaolog) capped and poly(A) tailed mRNAs encoding Flag-Setdb1 and Flag-C1243A were produced by *in vitro* transcription using HiScibe T7 ARCA mRNA kit (with tailing) from New England Biolabs (E2060S). The templates for *in vitro* transcription were generated by cloning *Flag-Setdb1* and *Flag-C1243A* cDNAs, respectively, in *pBluescript KS* (see S7 Fig for cloning strategy). The C1243A mutation was introduced by PCR. Primers used for molecular cloning are listed in S1 Table. All plasmid constructs were confirmed by DNA sequencing.

### Histological analysis and immunohistochemistry

Ovaries were collected and fixed in formalin overnight, processed, and embedded in paraffin by the Pathology Core Services Facility at MDACC using standard protocols. Ovaries were serially sectioned at 5 μm and stained with hematoxylin and eosine (H&E) or with periodic acid-Schiff (PAS)-hematoxylin. For IHC analysis, paraffin sections were deparaffinized and hydrated in xylene followed by 100% and 95% ethanol. Endogenous peroxidase activity was blocked with 3% H_2_O_2_ in water for 10 min. Antigen retrieve was done with 10 mM Citrate Buffer pH 6.0 in a microwave oven for 3 min. After blocking slides with Biocare Blocking Reagent (BS966M) for 10 min, slides were incubated with respective primary antibodies (listed in S2 Table) for 1 hour at room temperature. After incubating with appropriate horseradish peroxidase (HRP)-conjugated secondary antibodies (indicated in S2 Table) for 30 minutes at room temperature, slides were incubated with DAB monitoring staining development for viewing.

### Immunofluorescence

Isolated oocytes were washed in PBS containing 1% polyvinylpyrrolidine (PVP), fixed in 3.7% paraformaldehyde in PBS for 30 min, permeabilized for 15 min in 0.1% Triton X-100 in PBS, and then stained with respective primary antibodies (listed in S2 Table) overnight at 4°C. After washing three times with PBS containing 1mg/ml BSA, the oocytes were incubated for 1 hour with appropriate secondary antibodies conjugated to Fluorescein Isothiocyanate (FITC), Texas Red or Alexa Fluor 488 (indicated in S2 Table), followed by incubation with DAPI.

### Western blot

Western blot analysis of GV oocytes or ES cells was performed using standard procedures. GV oocytes were collected, washed in PBS containing 1% PVP, and boiled in sodium dodecyl sulfate (SDS) sample buffer. For comparisons, the same numbers of oocytes were used to assure equal loading. ES cells were lyzed in lysis buffer (20 mM Tris-HCl pH7.9, 25% glycerol, 150 mM NaCl, 1.5 mM MgCl_2_, 0.1% NP-40, 0.2 mM EDTA, and 0.5 mM DTT) supplemented with protease inhibitor cocktail (1861279, Nalgene) and phosphatase inhibitor cocktail (78427, Nalgene). The cells were then sonicated, centrifuged, and the supernatants were measured for protein concentrations using a protein assay kit (500–0116, Bio-Rad) and boiled in SDS sample buffer. For comparisons, equal amount (25 μg) of total proteins were loaded. The blots were probed with respective primary antibodies (listed in S2 Table) by overnight incubation at 4°C, followed by 1-hour incubation at room temperature with appropriate HRP-conjugated secondary antibodies (indicated in S2 Table). Protein bands were detected by Western Lightning ECL Pro detection reagent (NEL121001EA, PerkinElmer).

### qRT-PCR

Total RNA was extracted from 50–100 GV oocytes using the PicoPure RNA Isolation Kit (Life Technologies) according to the manufacturer's instruction, followed by reverse transcription (RT) using Superscript RT kit (Bio-Rad) to generate cDNA libraries. qRT-PCR was performed using iTaq Universal SYBR Green Supermix with ABI 7900 Real-Time PCR system (Applied Biosystems) using primers (listed in S1 Table) for the following genes and transposons: *Setdb1* (NM_1163641), *Cdc14b* (NM_172587), *Cdc25b* (NM_023117), *Bub1b* (NM_009773), *Ppp2cb* (NM_017374), *Cdk1* (NM_007659), *Ccnb1* (NM_172301), *Wee2* (NM_201370), *Fzr1* (NM_019757), *IAP*, *MTA*, *MusD*, and *Line1*.

### ChIP-qPCR analysis

ChIP assay was performed as previously described [36], using rabbit polyclonal antibodies to Setdb1 and H3K9me3 or normal rabbit IgG as negative control (see S2 Table for information about the antibodies). Briefly, *Setdb1*^*2lox/1lox*^ mouse ES cells transfected with *pCAG-Cre-ERT2* [28], as well as WT ES cells, were treated with 2 μM of 4-OHT for 4 days, and the treated cells (referred to as *Setdb1* KO and WT ES cells, respectively) were fixed with freshly prepared 1% paraformaldehyde for 10 min at room temperature. The cells were harvested and their nuclei extracted, lyzed, and sonicated. The samples were immunoprecipitated with 8 μg of Setdb1, H3K9me3, or normal IgG antibodies. The eluted protein:DNA complex was reverse-crosslinked at 65°C overnight. DNA was recovered after proteinase and RNase A treatment and then analyzed by real-time PCR using primers for the *Cdc14b* locus (listed in S1 Table).

### Statistical analysis

Statistical comparisons between samples were made using unpaired t-test or one-way ANOVA, and P < 0.05 was considered statistically significant.

## Supporting Information

S1 Fig Oocyte-specific deletion of *Setdb1*. **(A)** Schematic diagrams of the *Setdb1* alleles. Exons are shown as black bars. Exon 16, flanked by *loxP* sites (shown as triangles) in the conditional allele, encodes part of the catalytic bifurcated SET domain. The locations of the primers used for genotyping (F1, F2, and R1) are indicated. (**B)** Mating scheme used to produce *Setdb1* knockout (KO) and control mice. **(C)** Representative PCR genotyping results using tail-tip genomic DNA. For each sample, the left lane (lane 1, 3, 5, or 7) is *Cre* PCR, and the right lane (lane 2, 4, 6, or 8) is *Setdb1* allele PCR.
(PDF)

S2 Fig Decease in H3K9me2 in *Setdb1* KO growing oocytes. Ovarian sections of 2-month-old control and *Setdb1* KO mice were analyzed by immunohistochemistry (IHC) for H3K9me2 **(A)**, H3K9me1, H3K9me3 **(B)**, or H3K4me2 **(C)**, as indicated. Representative staining patterns of growing oocytes are shown, and their nuclei are indicated by arrows. Scale bars, 50 μm.
(PDF)

S3 Fig Setdb1 deficiency has no effect on oocyte growth. **(A)** Periodic acid-Schiff (PAS)-hematoxylin staining showing the histological features of ovaries from 2-month old control and *Setdb1* KO mice. CL, corpus luteum. Scale bar, 500 μm. **(B)** Quantification of follicles from control and *Setdb1* KO ovaries. Ovarian sections were examined by microscopy, and follicles of various stages were determined by morphology and counted. The data are presented as the mean ± SEM of 8 ovarian sections from 2 mice for each genotype. (**C**) Representative bright-field microscope images of control and *Setdb1* KO fully-grown GV oocytes showing no difference in morphology. Arrows indicate the prominent nucleoli characteristic of GV oocytes. Scale bar, 50 μm. **(D)** The numbers of fully-grown GV oocytes harvested from the ovaries of control and *Setdb1* KO mice are presented as the mean ± SEM (data from 5 control and 6 *Setdb1* KO mice).
(PDF)

S4 Fig Setdb1 binding and H3K9me3 enrichment in meiosis genes in mouse ES cells. Shown are genome browser screenshots of the *Cdc14b*, *Cdc25b*, *Bub1b*, and *Ppp2cb* loci showing Setdb1 and H3K9me3 ChIP-Seq data in mouse ES cells (from Bilodeau et al. 2009 [30]).
(PDF)

S5 Fig MG132 improves GVBD rate of Setdb1 KO oocytes. Control and *Setdb1* KO GV oocytes were collected in M2 medium supplemented with 200 M of IBMX so as to prevent oocytes from undergoing GVBD. *Setdb1* KO oocytes were treated with DMSO or MG132 (10 μM) for 4 hours. After washing, control and *Setdb1* KO oocytes were cultured in IBMX-free M16 medium for 2 hours. **(A)** Representative bright-field microscope images of control oocytes and *Setdb1* KO oocytes treated with or without MG132. Arrows indicate the prominent nucleoli characteristic of GV oocytes. Scale bars, 50 μm. **(B)** The percentages of GV, GVBD/MI, and abnormal oocytes. The numbers of oocytes analyzed are indicated.
(PDF)

S6 Fig Effect of *Cdc14b* knockdown on spindle phenotype during MI. GV oocytes were harvested from control and *Setdb1* KO mice. *Setdb1* KO oocytes were microinjected with either control siRNA or *Cdc14b* siRNA. The injected oocytes, as well as control GV oocytes, were incubated in IBMX-containing medium for 24 hours to allow siRNA-mediated *Cdc14b* depletion to occur while maintaining GV arrest and, following IBMX washout, were allowed to mature *in vitro* for another 6 hours. Oocytes were immunostained for α-tubulin (green) and DNA (blue) to examine spindle and chromosome structures. **(A)** Representative IF images showing MI oocytes with normal and abnormal spindle structures. **(B)** Percentages of MI oocytes with spindle defects in the indicated groups. The total number of MI oocytes examined were: 32 control, 30 *Setdb1* KO injected with control siRNA, and 34 *Setdb1* KO injected with *Cdc14b* siRNA. Statistical comparisons were made using one-way ANOVA. *P < 0.05; **P < 0.01.
(PDF)

S7 Fig Plasmid constructs used as templates for *in vitro* transcription for the production of mRNAs encoding Flag-tagged WT Setdb1 or catalytically inactive Setdb1 (C1243A). *Flag-Setdb1* or *Flag-C1243A* cDNA was inserted into the *Spe*I-*EcoR*I sites of *pBluescript KS*. The constructs were linearized with *Sal*I digestion before being used for *in vitro* transcription. The location of the C1243A point mutation is indicated.
(PDF)

S1 Table PCR primers. All primers used in this study are listed, including their sequences and applications.
(PDF)

S2 Table Antibodies. All antibodies used in this study are listed, including the vendors, catalogue numbers, dilutions, and applications.
(PDF)
