# Supplementary figures and images for: Maternal Setdb1 Is Required for Meiotic Progression and Preimplantation Development in Mouse
Source: PLoS Genet. 2016 Apr 12;12(4):e1005970. doi: 10.1371/journal.pgen.1005970 (PMC4829257; doi:10.1371/journal.pgen.1005970)

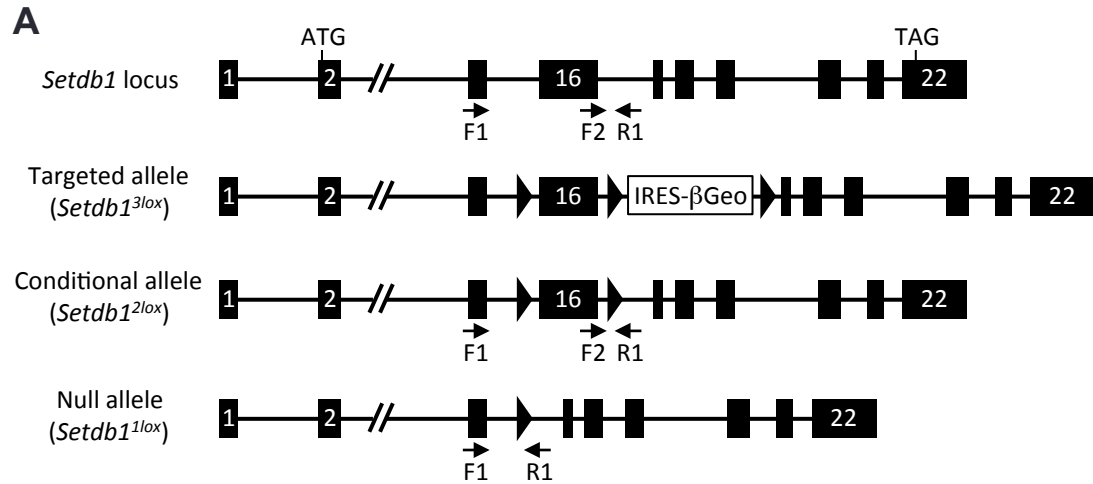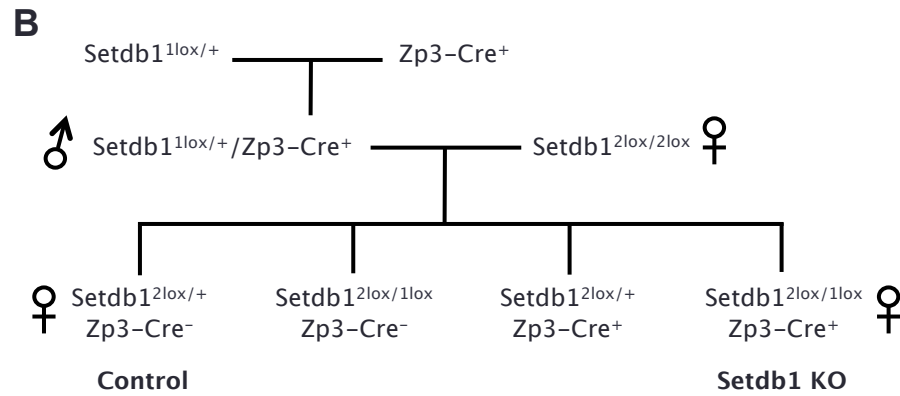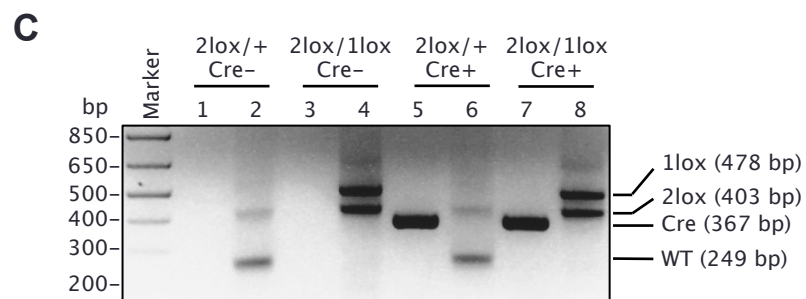

Supplement: S1 Fig — (A) Schematic diagrams of the Setdb1 alleles. Exons are shown as black bars. Exon 16, flanked by loxP sites (shown as triangles) in the conditional allele, encodes part of the catalytic bifurcated SET domain. The locations of the primers used for genotyping (F1, F2, and R1) are indicated. (B) Mating scheme used to produce Setdb1 knockout (KO) and control mice. (C) Representative PCR genotyping results using tail-tip genomic DNA. For each sample, the left lane (lane 1, 3, 5, or 7) is Cre PCR, and the right lane (lane 2, 4, 6, or 8) is Setdb1 allele PCR. (PDF) [file pgen.1005970.s001.pdf]

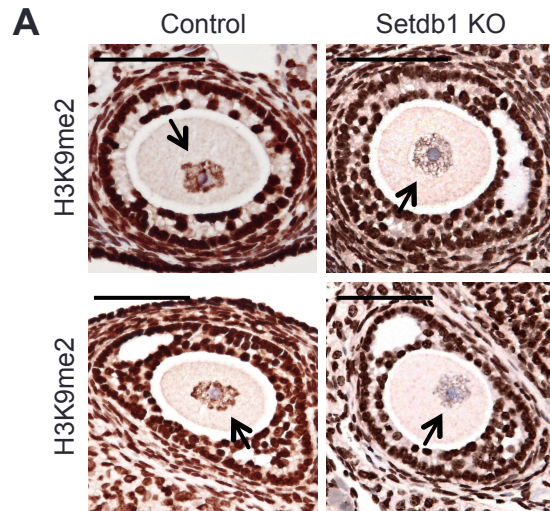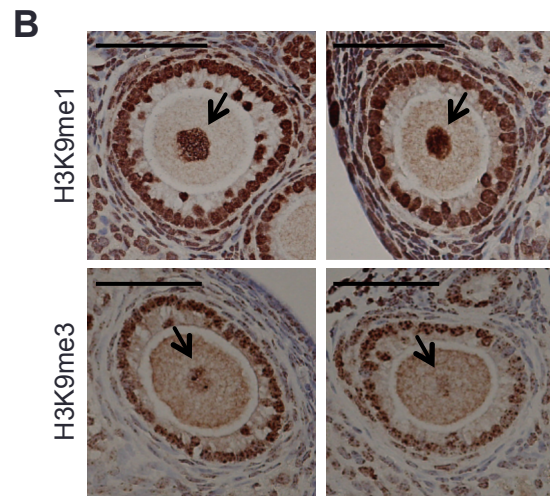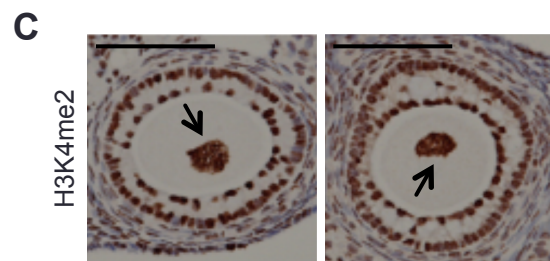

Supplement: S2 Fig — Ovarian sections of 2-month-old control and Setdb1 KO mice were analyzed by immunohistochemistry (IHC) for H3K9me2 (A), H3K9me1, H3K9me3 (B), or H3K4me2 (C), as indicated. Representative staining patterns of growing oocytes are shown, and their nuclei are indicated by arrows. Scale bars, 50 μm. (PDF) [file pgen.1005970.s002.pdf]

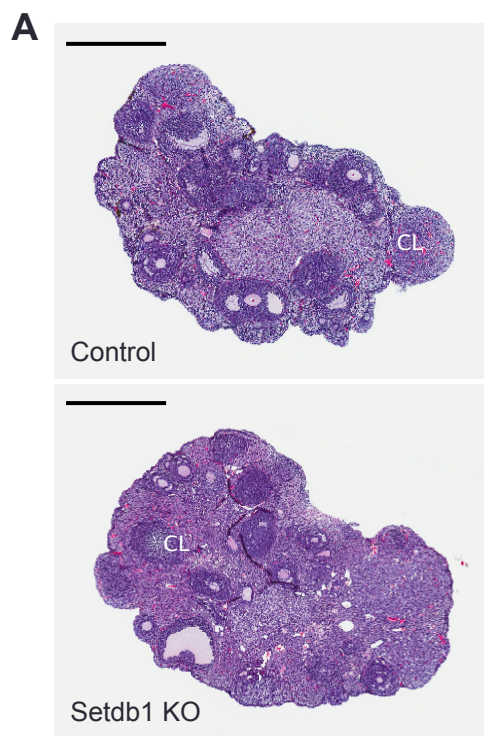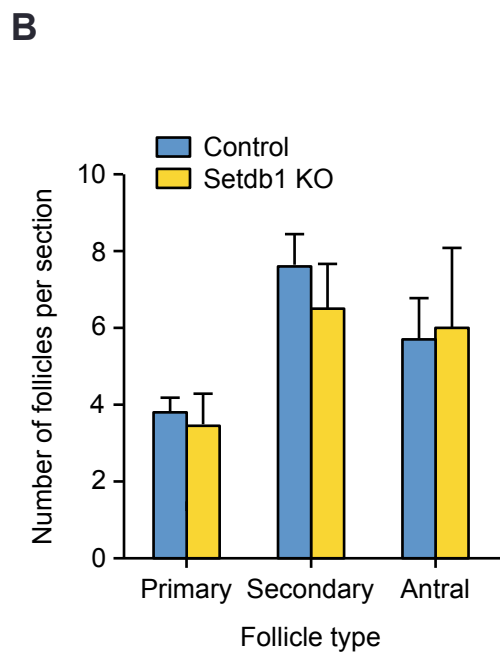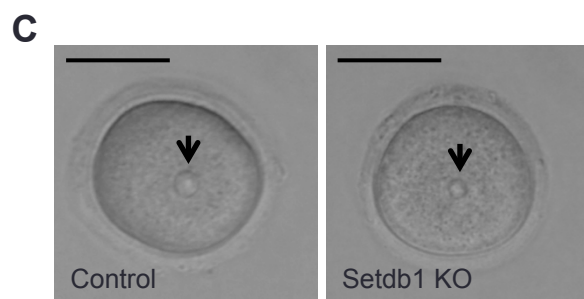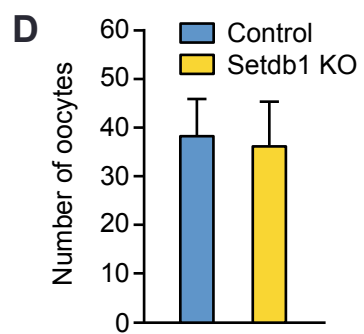

Supplement: S3 Fig — (A) Periodic acid-Schiff (PAS)-hematoxylin staining showing the histological features of ovaries from 2-month old control and Setdb1 KO mice. CL, corpus luteum. Scale bar, 500 μm. (B) Quantification of follicles from control and Setdb1 KO ovaries. Ovarian sections were examined by microscopy, and follicles of various stages were determined by morphology and counted. The data are presented as the mean ± SEM of 8 ovarian sections from 2 mice for each genotype. (C) Representative bright-field microscope images of control and Setdb1 KO fully-grown GV oocytes showing no difference in morphology. Arrows indicate the prominent nucleoli characteristic of GV oocytes. Scale bar, 50 μm. (D) The numbers of fully-grown GV oocytes harvested from the ovaries of control and Setdb1 KO mice are presented as the mean ± SEM (data from 5 control and 6 Setdb1 KO mice). (PDF) [file pgen.1005970.s003.pdf]

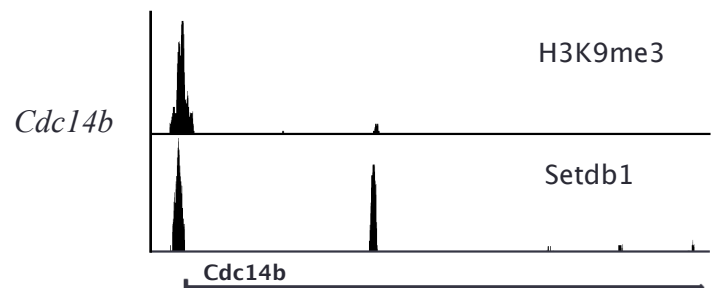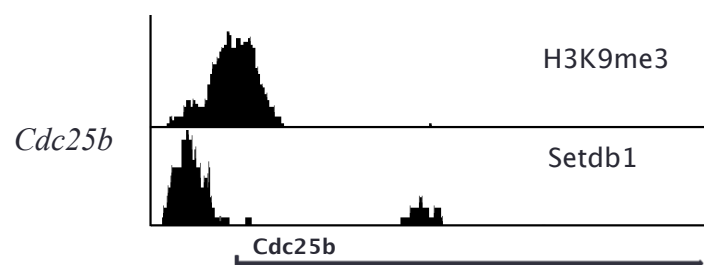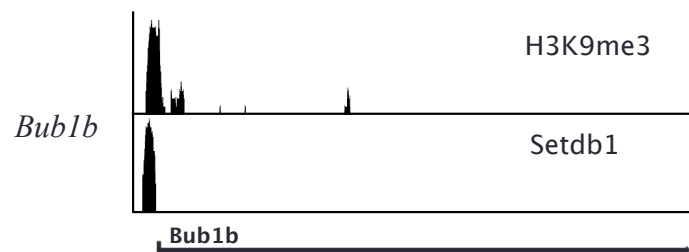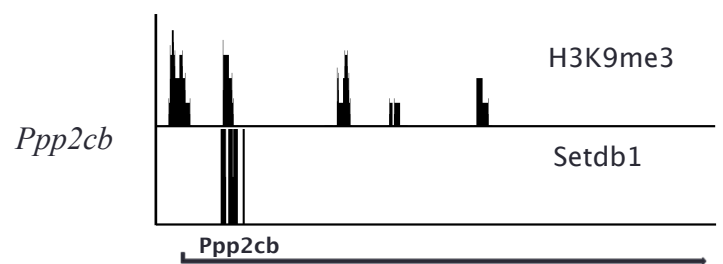

20 Kb

Supplement: S4 Fig — Shown are genome browser screenshots of the Cdc14b, Cdc25b, Bub1b, and Ppp2cb loci showing Setdb1 and H3K9me3 ChIP-Seq data in mouse ES cells (from Bilodeau et al. 2009 [30]). (PDF) [file pgen.1005970.s004.pdf]

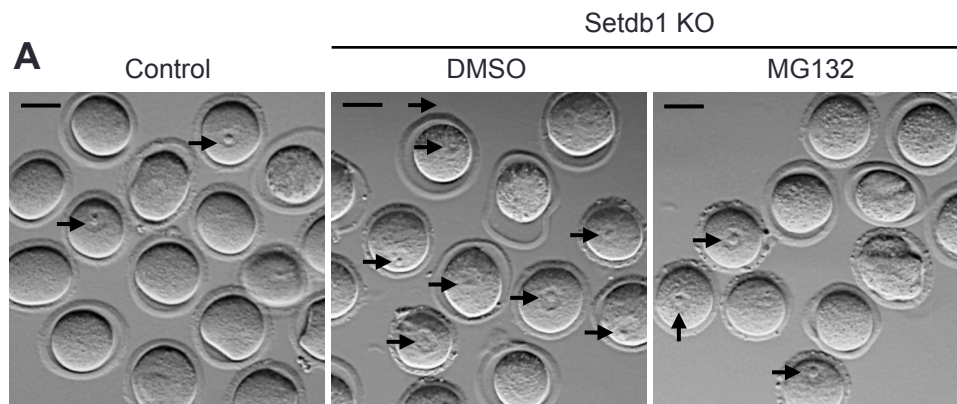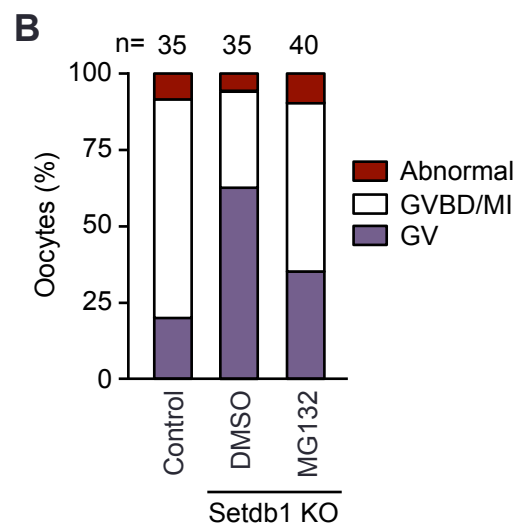

Supplement: S5 Fig — Control and Setdb1 KO GV oocytes were collected in M2 medium supplemented with 200 M of IBMX so as to prevent oocytes from undergoing GVBD. Setdb1 KO oocytes were treated with DMSO or MG132 (10 μM) for 4 hours. After washing, control and Setdb1 KO oocytes were cultured in IBMX-free M16 medium for 2 hours. (A) Representative bright-field microscope images of control oocytes and Setdb1 KO oocytes treated with or without MG132. Arrows indicate the prominent nucleoli characteristic of GV oocytes. Scale bars, 50 μm. (B) The percentages of GV, GVBD/MI, and abnormal oocytes. The numbers of oocytes analyzed are indicated. (PDF) [file pgen.1005970.s005.pdf]

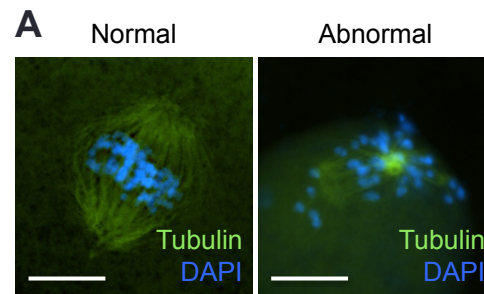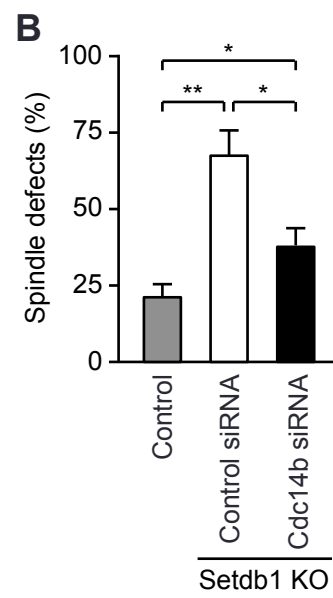

Supplement: S6 Fig — GV oocytes were harvested from control and Setdb1 KO mice. Setdb1 KO oocytes were microinjected with either control siRNA or Cdc14b siRNA. The injected oocytes, as well as control GV oocytes, were incubated in IBMX-containing medium for 24 hours to allow siRNA-mediated Cdc14b depletion to occur while maintaining GV arrest and, following IBMX washout, were allowed to mature in vitro for another 6 hours. Oocytes were immunostained for α-tubulin (green) and DNA (blue) to examine spindle and chromosome structures. (A) Representative IF images showing MI oocytes with normal and abnormal spindle structures. (B) Percentages of MI oocytes with spindle defects in the indicated groups. The total number of MI oocytes examined were: 32 control, 30 Setdb1 KO injected with control siRNA, and 34 Setdb1 KO injected with Cdc14b siRNA. Statistical comparisons were made using one-way ANOVA. *P < 0.05; **P < 0.01. (PDF) [file pgen.1005970.s006.pdf]

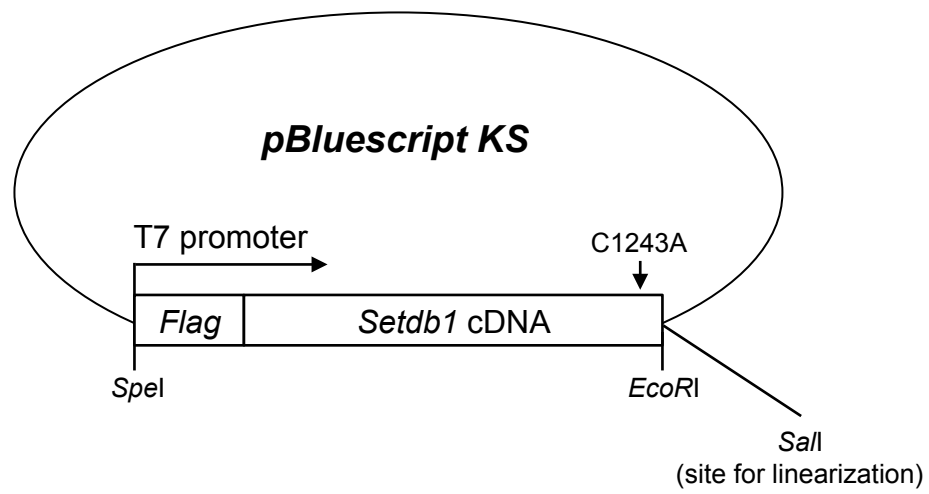

Supplement: S7 Fig — Flag-Setdb1 or Flag-C1243A cDNA was inserted into the SpeI-EcoRI sites of pBluescript KS. The constructs were linearized with SalI digestion before being used for in vitro transcription. The location of the C1243A point mutation is indicated. (PDF) [file pgen.1005970.s007.pdf]
