# Supplementary material for: Maternal Setdb1 Is Required for Meiotic Progression and Preimplantation Development in Mouse
Source: PLoS Genet. 2016 Apr 12;12(4):e1005970. doi: 10.1371/journal.pgen.1005970 (PMC4829257; doi:10.1371/journal.pgen.1005970)
Supplement: S1 Table — All primers used in this study are listed, including their sequences and applications. (PDF) [file pgen.1005970.s008.pdf]

S1 Table. PCR Primers

Primers	Sequences (5' – 3')	Applications
Setdb1-F1<br>Setdb1-R1<br>Setdb1-F2	GTATCCGCTGCTTGGATGATATTG<br>CATCTTGTTTCGGTTCTCAGGGTC<br>GGTCCGTGAAGCACAGAGCTCACG	*Setdb1* genotyping<br>*WT:* 249 bp,<br>*Setdb1*^{1lox}: 478 bp<br>*Setdb1*^{2lox}: 403 bp
Cre-F<br>Cre-R	GCAAGTTGAATAACCGGAAATGG<br>GCAATTTCGGCTATACGTAACAG	*Cre* genotyping<br>Cre: 367bp
IAP-F<br>IAP-R	AAGCAGCAATCACCCACTTTGG<br>CAATCATTAGATGTGGCTGCCAAG	*IAP* qRT-PCR
Line1-F<br>Line1-R	GAGACATAACAACAGATCCTGA<br>AACTTTGGTACCTGGTATCTG	*Line1* qRT-PCR
MusD-F<br>MusD-R	GTGGTATCTCAGGAGGAGTGCC<br>GGGCAGCTCCTCTATCTGAGTG	*MusD* qRT-PCR
MTA-F<br>MTA-R	ATGTCTTGGGGAGGACTGTGGAAG<br>AGCCCCAGCTAACCAGAACTACAG	*MTA* qRT-PCR
Setdb1-F<br>Setdb1-R	CTTGGGAGGACATAGAAGATAG<br>TTTTGTCATCCAGAAAGAGG	*Setdb1* qRT-PCR
Cdc14b-F<br>Cdc14b-R	CAGCCTATATTCCTTTCAGAG<br>TACTGCATTGCCTTCTTTAC	*Cdc14b* qRT-PCR
Cdc25b-F<br>Cdc25b-R	GAGTGATTTAAAGGATGACGAG<br>ATGATGAGATCCTGTTCCTC	*Cdc25b* qRT-PCR
Bub1b-F<br>Bub1b-R	TTGGACAGAACAGAACTACC<br>AATGTCCCAATTTGATCCAG	*Bub1b* qRT-PCR
Ppp2cb-F<br>Ppp2cb-R	ACAGCTTTAGTAGATGGACAG<br>CATAAGAGATCACACATTGGG	*Ppp2cb* qRT-PCR
Cdk1-F<br>Cdk1-R	AGAAGGTACTTACGGTGTGGT<br>GAGAGATTTCCCGAATTGCAGT	*Cdk1* qRT-PCR
Ccnb1-F<br>Ccnb1-R	GCGTGTGCCTGTGACAGTTA<br>CCTAGCGTTTTTGCTTCCCTT	*Ccnb1* qRT-PCR
Wee2-F<br>Wee2-R	AGAGAATTACCAACACCTCC<br>GATCATGTTCTTGAGTAGACC	*Wee2* qRT-PCR
Fzr1-F<br>Fzr1-R	GAAGGGGACTCAGTGACTTCC<br>CACGGCTACCAGATGACAACT	*Fzr1* qRT-PCR
Cdc14b-R1-F<br>Cdc14b-R1-R	GGTCGCCAGCACGCTTTCTGCCCG<br>AGGAGGGATGTTCCGTGGAGCTTC	*Cdc14b* R1 region<br>ChIP-qPCR
Cdc14b-R2-F<br>Cdc14b-R2-R	AGCAGACACACAGAATTCCACTTG<br>GTAAGTAGCAAAGGGTCTTATAGC	*Cdc14b* R2 region<br>ChIP-qPCR
C1243A-F<br>C1243A-R	CAATCACAGTgcCAGCCCCAACCTGTTTGTC<br>GTTGGGGCTGgcACTGTGATTGAGGTAGCG	Primers for Setdb1<br>C1243A mutation
