# Supplementary material for: Maternal Setdb1 Is Required for Meiotic Progression and Preimplantation Development in Mouse
Source: PLoS Genet. 2016 Apr 12;12(4):e1005970. doi: 10.1371/journal.pgen.1005970 (PMC4829257; doi:10.1371/journal.pgen.1005970)
Supplement: S2 Table — All antibodies used in this study are listed, including the vendors, catalogue numbers, dilutions, and applications. (PDF) [file pgen.1005970.s009.pdf]

S2 Table. Antibodies

Antibody	Vendor, cat. No.	Dilution	Secondary antibody	Application
Setdb1	Cell Applications, CP10377	1:500	Goat anti-mouse-HRP	WB
Setdb1	Santa Cruz, sc-66884	NA	NA	ChIP
Cdc14b	Bioss, bs-7608R	1:100	Goat anti-rabbit-TxRed	IF
Cdc14b	Abcam, ab203675	1:500	Goat anti-rabbit-HRP	WB
Cyclin B1	Cell Signaling, 4135	1:1,000	Goat anti-mouse-HRP	WB
γ-H2AX	Cell Signaling, 9718	1:100	Goat anti-rabbit-TxRed	IF
α-tubulin	Cell Signaling, 2144	1:1,000	Goat anti-rabbit-HRP	WB
α-tubulin	Cell Signaling. 8058	1:100	Goat anti-mouse-Alexa Fluor488	IF
β-actin	Sigma, A5441	1:1000	Goat anti-mouse-HRP	WB
Flag tag	Sigma F3165	1:200	Goat anti-mouse-FITC	IF
H3K9me1	Cell Signaling 14186	1:100	Goat anti-rabbit-HRP	IHC
H3K9me1	Cell Signaling 14186	1:100	Goat anti-rabbit -FITC	IF
H3K9me2	Cell Signaling 9753	1:100	Goat anti-rabbit-HRP	IHC
H3K9me2	Cell Signaling 9753	1:100	Goat anti-rabbit -FITC	IF
H3K9me3	Millipore 07-442	1:100	Goat anti-rabbit-HRP	IHC
H3K9me3	Millipore 07-442	1:100	Goat anti-rabbit -FITC	IF
H3K9me3	Abcam ab8898	NA	NA	ChIP
H3K4me2	Cell Signaling 9725	1:500	Goat anti-rabbit-HRP	IHC
Rabbit IgG	Cell Signaling 2729	NA	NA	ChIP

WB, Western blot; ChIP, chromatin immunoprecipitation; IF, immunofluorescence; IHC, immunohistochemistry; HRP, horseradish peroxidase; TxRed, Texas Red; FITC, Fluorescein Isothiocyanate; NA, not applicable (for each ChIP, 8 μg of antibody were used).
